# Supplementary material for: Effective polyploidy causes phenotypic delay and influences bacterial evolvability
Source: PLoS Biol. 2018 Feb 22;16(2):e2004644. doi: 10.1371/journal.pbio.2004644 (PMC5839593; doi:10.1371/journal.pbio.2004644)
Supplement: S1 Text — (PDF) [file pbio.2004644.s001.pdf]

Effective polyploidy causes phenotypic delay and influences bacterial evolvability

S1 Text

Lei Sun, Helen K. Alexander, Balazs Bogos, Daniel J. Kiviet,

Martin Ackermann, Sebastian Bonhoeffer

1 Detailed recombineering protocol

Introduction: The purpose of this document is to describe the recombineering protocol in detail to help other researchers scrutinise our work and reproduce it if necessary. The strains and ssDNA oligonucleotides used are listed in Tables S2 and S3. The technicalities of recombineering have been described extensively in previous studies, from which this protocol is optimised[1;2]. In brief, recombineering relies on the cell's own DNA replication machinery for point-specific mutagenesis, mimicking mutations arising during replication in dividing cells. The target site for recombineering mutagenesis on a bacterial chromosome must first be exposed by an open replication fork as single-strand DNA. The exogenous ssDNA oligonucleotide then binds complementarily to its exposed target like an Okazaki-fragment on the lagging strand, albeit with a few mismatches that carry the mutations. The methyl-directed mismatch repair corrects individual mismatches with high efficiency but fails to fix consecutive mismatches. Two synonymous mutations were therefore added on each side of the actual non-synonymous mutation to prevent it from being repaired and thus boost the mutagenesis efficiency. These mismatches form a heteroduplex double-strand chromosomal DNA at the target site, resembling natural mutations in bacteria. After one additional round of DNA replication this heteroduplex chromosome segregates into one wild-type and one mutant chromosome. Since the exogenous ssDNA oligonucleotide integrates into the lagging strand, its sequence orientation depends on the target gene relative to the replication origin. All three antibiotic resistance target genes in this study required anti-sense ssDNA for mutagenesis, as this is the only orientation that allows efficient recombineering. Given that sense mRNA is transcribed reverse-complementarily

from the anti-sense DNA strand, mutating the anti-sense DNA strand should allow immediate production of mutant mRNA, as opposed to mutating the sense DNA strand. There is indeed some evidence that mutant mRNA can be transcribed from mismatched heteroduplexes[17].

When induced by heat, the recombineering plasmid pSIM6 expresses proteins that facilitate mutagenesis by, for instance, binding to the exogenous oligonucleotide and delaying the otherwise rapid degradation of cytosolic ssDNA. Recombineering efficiency in cells grown from minimal media is typically orders of magnitude lower than in rich media, due to poor ssDNA uptake and fewer open replication forks for ssDNA integration[2]. Manipulating growth rate with sub-lethal antibiotics such as ciprofloxacin may reduce the overall growth rate of cell population but cause cell filamentation, thereby promoting polyploidy instead of reducing it[3]. Due to these technical limitations, instead of manipulating bacterial ploidy through choice of media, we kept media consistent and quantified ploidy by using the lacZ-reporter assay.

The precise day-by-day experimental protocol is as follows.

Day 1: An *E. coli* MG1655 strain containing the pSIM6 recombineering plasmid was streaked from frozen stock onto LB agar plates with 100 μg/ml ampicillin and incubated overnight at 30°C until colonies became visible. The pSIM6 plasmid was maintained by ampicillin and kept at below 32°C except during the heat-activation on Day 3.

Day 2: A colony was picked to inoculate 5 ml overnight culture in LB supplemented with 100 μg/ml ampicillin and incubated at 30°C. Fresh antibiotic plates were prepared for phenotypic selection of resistance mutations (rifampicin, streptomycin or nalidixic acid). Liquid LB was prepared and incubated overnight in 30°C to pre-warm for day 3. Our LB contained only 5 g/L sodium chloride, since the other commonly used concentration of 10 g/L was previously reported to be suboptimal for recombineering[1]. Sterile de-ionized Milli-Q water was prepared and incubated at 4°C overnight in the fridge.

Day 3: 1.5 ml of fully-grown overnight culture was transferred into 120 ml pre-warmed LB supplemented with 100 μg/ml ampicillin in a large Erlenmeyer flask. Each flask was enough for about 10 electroporations. The flask culture was incubated at 30°C with shaking at 220 rpm until reaching an OD600 of $\sim$0.3 after approximately 2 hours. The flask was placed in a 43°C water bath with 50-100 rpm shaking for 10 minutes to induce expression of the recombineering proteins on pSIM6. Immediately after heat shock, the flask culture was rapidly chilled by gentle

swirling in an ice-water mix and left on ice for at least 5 min. The chilled culture was aliquoted into 50-ml Falcon tubes. Cells were centrifuged down at 4000 G for 5 minutes at 4°C. The LB was removed and cells resuspended by pipetting gently up and down in ice-cold Milli-Q water. Cells were washed in ice-cold water three times with at least 3 ml water each time. During the wash it was crucial to remove as much residual liquid as possible after each centrifugation, especially when removing the LB. The pellet from each Falcon tube, concentrated down from 50 ml of early-exponential phase bacterial culture, was finally resuspended in 250 μl of ice-cold water after the third wash. From this cell suspension we aliquoted and mixed every 50 μl of cells with 1 μl of 5 μM ssDNA oligo ($\sim$200 ng for a 90 base oligo) in a clean Eppendorf tube. The cell-DNA mix was then transferred into a 1-mm gap electroporation cuvette chilled by ice-cold water.

Cell-DNA mix was electroporated at 1.8 kV/mm with time constant around 5.0 ms. After each electroporation, 1 ml fresh LB was immediately added into the cuvette, before proceeding to the next cuvette. We had in each experiment typically six replicates. We also included two controls: (i) cell sample diluted directly into fresh LB without electroporation; and (ii) cell sample electroporated without adding ssDNA. All electroporated samples as well as the non-electroporated control were incubated for 30 min at 30°C without shaking for cells to recover. The entire sample from each cuvette was transferred to a clean Eppendorf tube and centrifuged down at 4000 G in a table-top centrifuge at room temperature for 3 minutes, then resuspended in 1 ml fresh LB. Cell density in these 1-ml cultures without electroporation was determined (by serial dilution and plating from the non-electroporated control) to be around 10^8 to 10^9 per ml. This is because every 1-ml of resuspended culture contained cells from $\sim$10 ml of early-exponential phase culture that started from a 80-fold diluted stationary phase culture (with a density of 10^9 cells per ml) and grew for $\sim$2 hours at 30°C. Electroporation reduces the viable cell density by about one order of magnitude, yielding 10^7-10^8 viable cells in each 1-ml culture. This allows up to 6 cell divisions before reaching full density. All the resuspended 1-ml cultures were incubated at 30°C with 220 rpm shaking. To maintain continuous growth, cultures were diluted 100-fold into 1 ml fresh LB after 6 hours and 24 hours. In our experiments the cells first grew slowly, reaching 3-5 generations after 6h. After the first transfer at 6h, they would reach 10-13 generations at 24h (for an example, see S10 Fig). After the second transfer at 24h, they would reach $\sim$20 generations at 48h.

We sampled the cultures hourly for the first 10 hours (Day 3), then at 24h (Day 4) and 48h (Day 5) post-electroporation. For phenotyping, at each time point we sampled 20 μl of

culture, which was roughly 2% of the initial volume. The samples were diluted in PBS before plating on non-selective media (for determining total cell density and genotypic mutant frequency) or selective media plates (for determining phenotypic mutant frequency). The dilution factor was adjusted accordingly. At the very first sampling for example, 90 μl of 10,000-fold diluted culture could yield a few hundred CFUs on non-selective media that allowed optimal counting on a normal 90 mm petri dish. This CFU-count from non-selective media was used to determine the bacterial population size in the culture, based on the known dilution factor and plating volume. The population size at each sampling time point was then compared to the population size sampled at time zero (right after the 30-min incubation following electroporation) to determine the number of population doublings, or bacterial generations, elapsed. For selective plating from the first sampling time point, 90 μl of 10-fold diluted culture may or may not yield even a single phenotypic mutant due to phenotypic delay. At later time points we diluted the samples more as cell density increased and genotypic mutants eventually developed their phenotype. We recommend testing the exact dilution factor and plating volume in smaller pilot experiments beforehand. (For the resistance mutations, we performed five pilot/prior experiments that showed consistent patterns of phenotypic delay, before we proceeded to the experiments reported here, with more sampling time points, samples and replicates per experiment. Similarly, for the lacZ-reporter assay, we established the method with two pilot experiments beforehand.)

When working with the *lacZ*-reporter assay we used non-selective media plates containing 1 mM IPTG and 40 μg/ml X-gal. As visible colonies formed after overnight incubation, the plates were left at 4° for about a week for the blue colour to fully develop. Genotypic mutants could be directly visualised by colonies that contain blue colour. Phenotypic *lacZ*-mutants were scored on M9 agar plates with 0.4% lactose as carbon source.

All plates were incubated at 30°C. The non-selective media plates were incubated overnight and used to quantify the population size of the bacteria as well as assessing the frequency of genotypic mutants (see Day 4). Selective plates were incubated up to 48 hours, thus giving the mutants sufficient time to grow in the presence of selection before counting the phenotypic mutant colonies. At the same sampling time points we also plated cells from the two controls onto selective media. We observed no colonies resulting from either spontaneous mutations or drug-tolerance in these controls.

Day 4: CFU on non-selective plates incubated from Day 3 were counted to assess the population size of the bacteria at each sampling time point. The change in bacterial cell density over time was translated into growth expressed in number of bacterial generations. After counting CFU, entire plates of colonies were replica-plated onto selective media plates. Since the MIC of fully-developed phenotypic mutants are at least ∼100 times higher than that of wild-type cells, colonies were replicated onto selective media with the drug concentration of 8x MIC rather than 2x MIC. The fraction of colonies that subsequently grew on selective media among all colonies from the same plate was recorded as the frequency of genotypic mutants (see Day 5). We replica-plated 100-1000 colonies from each replicate at each sampling time point to ensure a decent estimate of genotypic mutant frequency. The replicated plates were incubated at room temperature overnight as doing so allows sufficient growth for replicated mutant colonies but limits the growth of rare, spontaneous mutants in wild-type colonies.

Day 5: Visible patches of cells on replica-plated selective plates, grown from mutant colonies transferred from non-selective plates on Day 4, were counted. This number was divided by the total CFU on non-selective plates counted on Day 4, thereby obtaining the genotypic mutant frequency. Phenotypic mutant CFU on selective plates with cells sampled from Day 3 were counted after the 48-hour incubation. Phenotypic mutant frequency was calculated based on the CFU counts, the known plating volume and dilution factor. Phenotypic penetrance was calculated as the ratio between phenotypic mutant frequency and genotypic mutant frequency for each replicate from each sampling time point.

Finally, the 48-hour time point was sampled on Day 5. Procedures for scoring genotypic and phenotypic mutants, as described above, were repeated as required on the following days for the 24-hour and 48-hour samples.

2 Fluctuation tests

2.1 Detailed simulation methods

Spontaneous *de novo* mutations: We adopted the standard "Lea-Coulson" formulation[4;5] to model the appearance of mutants during culture growth. In time t_f, the wild-type population grows deterministically from size N_0 to $N_f = N_0 2^{t_f} = N_0 \mathrm{e}^{\beta t_f}$, where $\beta = \log 2$ is the exponential growth rate per unit time, with one time unit taken as the population doubling time. *De*

novo mutants appear according to a non-homogeneous Poisson process with instantaneous rate $\beta\mu N_0 e^{\beta t}$ at time t. The mutation rate μ to be estimated has the interpretation of mutations generated per wild-type cell division in this standard approach[6]. (Note that our μ corresponds to μ_β in certain key references[7;6].) We then applied the results[8;9] that, to a good approximation, the total number of mutation events occurring is Poisson-distributed with mean $\mu N_0(2^{t_f} - 1)$, and each clone descending from a *de novo* mutant has "developing time" (time remaining until t_f) exponentially distributed with mean $1/\beta = 1/\log 2$. We drew random values from these distributions to determine the number of mutant clones to simulate and for how long. To account for polyploidy, we deconstructed the mutation rate as $\mu = c\mu_c$ where c is ploidy and μ_c is mutation rate per target copy. We assumed that a mutation arises on a single chromosome within a cell, and neglected the chance (of order μ^2) that more than one chromosome mutates either simultaneously in one cell or later in the same mutant clone.

Unlike recombineering, which introduces mutations in the anti-sense DNA strand (see section 1), natural mutations may arise by a number of mechanisms. For instance, mutations immediately affecting both strands may arise when DNA double-strand breaks occur and introduce genome rearrangements such as inversions, insertions or deletions upon repair of the break. Mutations can also arise by damage or copying error affecting only a single strand. This results in a mismatch which, if not repaired, will lead to a fully fledged double-stranded mutation in one of the two descendant chromosomes only after another round of DNA replication. By assuming that all descendants of a *de novo* mutant are also mutants, the standard model implicitly supposes that mutations arise in double-stranded form. In order to have agreement with the standard model in the monoploid case, and thus focus our investigation on the effect of ploidy, we also assumed that mutations arise in double-stranded form in our simulation model. If mutations actually arise in single-stranded form, in most cases this will give equivalent results to our double-stranded polyploid model with adjusted parameter settings (see section 2.3 below).

Mutant clone dynamics: We tracked every cell in a mutant clone individually, including its chromosome composition and timepoints at which it is produced by division and in turn divides again. The interdivision time of each cell was either taken to be constant, or drawn independently at random from an exponential distribution. By assuming independence, we neglect any possible correlations of interdivision time between sister cells or across generations. Other interdivision time distributions could readily be implemented, but these two cases cap-

ture the extremes of variance between which realistic interdivision time distributions will lie. The exponential case corresponds to the standard Lea-Coulson formulation, in which the development of mutant clones follows a Yule process. A more complete investigation of interdivision time distributions in the context of fluctuation tests, though limited to monoploidy, has been undertaken by other authors[9;10], and our observation of similar estimated mutation rate for the two different interdivision time distributions (S2 Fig) is consistent with these previous studies.

Also as in the standard model, we assumed that the mutant has the same fitness as the wild-type in non-selective media, in the sense that expected population doubling time is equal. To achieve this, we fixed median interdivision time of mutant cells to one time unit (requiring interdivision time to be exactly one in the constant case, or exponentially distributed with rate β in the exponential case). Mutant clones were simulated until no more divisions before t_f occurred, then cells existing at time t_f were scored for phenotype according to their chromosome composition and assumed dominance.

Our method of tracking cell "lifetimes" computationally was in part inspired by a previous approach[9], and these authors' consideration of general interdivision time distributions, along with other extensions such as different growth rates of wild-type and mutant in non-selective media, has recently been implemented in an R package[10]. Our consideration of multiple chromosome copies here is (to the best of our knowledge) novel, and could potentially be integrated into R packages in the future.

Maximum likelihood estimation: The calculation of the likelihood under the standard model has been described previously[11;7]. In brief, for any value of the composite parameter $m = \mu(N_f - N_0)$ (representing the mean number of mutation events occurring during culture growth), the probability mass function of the number of mutants in a culture at the end of growth can readily be calculated by recursive equations (Eq. 8 in ref.[7]), which we implemented in R. We thereby computed the likelihood as a function of m, given the number of phenotypic mutants observed in each parallel culture. Numerical optimization yielded the maximum likelihood estimate of m. The endpoints of the 95% profile likelihood confidence interval were found by solving numerically for the values of m at the threshold for rejection in a likelihood ratio test at 5% significance level. Finally, the estimate of m was converted to an estimate of μ by dividing by $(N_f - N_0)$, assumed as usual to be a known and fixed value.

2.2 Mathematical explanation for the distribution of phenotypic mutants

While it has occasionally been recognized in the literature that the existence of multiple chromosome copies and resulting "segregation lag" could affect fluctuation analysis[12;13;14;15], the precise impact on the observed total mutant counts and resulting effects on mutation rate estimation under modern maximum likelihood methods have, to the best of our knowledge, never been elucidated. Our simulation study numerically illustrated the impact of effective polyploidy on mutation rate estimation. Here we provide a more detailed mathematical explanation of how effective polyploidy affects the distribution of the number of phenotypic mutants, thereby clarifying the numerical results.

2.2.1 Recessive mutations

When mutations are recessive, we observed numerically that the estimated mutation rate $\hat{\mu}$ was close to the actual per-copy mutation rate μ_c, regardless of ploidy, and that the distribution of mutant counts predicted by the standard model parameterized by $\hat{\mu}$ provided a good match to the observed distribution from simulations of polyploid populations. These observations can be explained mathematically.

After the appearance of a mutation in one out of c copies in a cell, according to our model of segregation there will be a delay of $n = \log_2 c$ cell divisions until the first homozygous mutant appears; all other descendants up to this time are heterozygotes or homozygous wild-type cells (S1 Fig). Since the mutation is recessive, no mutants are "visible" until homozygosity is achieved. (Recall that phenotypically wild-type cells are assumed to have no chance of dividing and forming visible colonies on selective plates in the fluctuation test.) After this lag, a phenotypic mutant lineage appears in which all further descendants are phenotypic mutants, regardless of ploidy. That is, the number of phenotypic mutants doubles in each subsequent generation, the same pattern as observed (without any lag) in a monoploid population.

Let T_{clone} denote the developing time of a randomly chosen mutant clone, i.e. the time between a *de novo* mutation arising in a single chromsome copy and the end time of culture growth (t_f) at which phenotypic mutants are counted. Since the timing of mutations is stochastic, T_{clone} is a random variable. The distribution of T_{clone} is approximately exponential with rate parameter $\beta = \log 2$ (ref.[8]). In our model, exactly n divisions are required to achieve homozygosity; however, the actual time between divisions can be variable. Thus the lag time

until a clone achieves homozygosity, say L, is in general a random variable, independent of T_{clone} and equal in distribution to the sum of n independent draws from the interdivision time distribution. A clone will produce visible descendants by t_f if and only if $T_{\text{clone}} \geq L$; in this case, we need to determine the number of visible descendants produced in the remaining time $T_{\text{clone}} - L$.

Firstly, we determine the probability that a randomly chosen clone produces visible descendants by t_f, i.e. $\Pr(T_{\text{clone}} \geq L)$. Recall that T_{clone} is approximately exponentially distributed, with

$$\Pr(T_{\text{clone}} \geq t) = \mathrm{e}^{-\beta t} \tag{1}$$

For constant interdivision time, the lag L is exactly equal to n time units. The probability of producing visible descendants is then simply:

$$\Pr(T_{\text{clone}} \geq n) = \mathrm{e}^{-\beta n} = 2^{-n} = 1/c$$

In fact, it can be shown (see below) that $\Pr(T_{\text{clone}} \geq L) = 1/c$ even for variable interdivision times, provided the population doubling time is fixed to one unit (i.e. equal fitness of mutant and wild-type). Recalling that the total number of mutant clones produced in the growing culture is approximately Poisson-distributed with mean $c\mu_c(N_f - N_0)$ (refs.[8;9]), and using the present result that each mutant clone (independently) produces visible descendants with probability $1/c$, the number of such "visible clones" is thus approximately Poisson-distributed with mean $\mu_c(N_f - N_0)$. Importantly, the number of visible clones is therefore independent of ploidy: the increase in mutational influx with c is exactly counterbalanced by the increase in segregation lag.

Secondly, we require the distribution of remaining developing time in clones once they cross their lag time. Recalling that T_{clone} is (approximately) exponentially distributed, however, we can simply apply the memoryless property:

$$\Pr(T_{\text{clone}} - L \geq t \,|\, T_{\text{clone}} - L \geq 0) = \Pr(T_{\text{clone}} \geq t)$$

In words, the remaining developing time after achieving homozygosity ($T_{\text{clone}} - L$), conditioned on homozygosity being reached before the end of the developing time ($T_{\text{clone}} - L \geq 0$), is equal in distribution to the total developing time of a randomly chosen clone (T_{clone}). In this remaining time, the mutant clone produces only homozygous mutant descendants, with a pattern of growth that is independent of ploidy. Therefore, provided a mutant clone does produce visible

descendants by time t_f, the number of these descendants is statistically no different to the monoploid case.

In summary, a ploidy-c population is expected to produce a Poisson-distributed number of "visible" clones with mean $\mu_c(N_f - N_0)$ by observation time t_f, each consisting of a number of phenotypic mutants equal in distribution to the monoploid case. (All other mutation events do not produce any phenotypic mutants by t_f and can thus be ignored.) The total number of phenotypic mutants counted is thus equal in distribution to a monoploid population with mutation rate μ_c in the standard model, explaining why we consistently estimate $\hat{\mu}$ close to μ_c, regardless of ploidy, and find that the standard model provides a good fit to the observed mutant count distribution across simulated parallel cultures. In the case of recessive mutations, then, effective polyploidy leaves no signature in fluctuation assay data.

Proof for variable interdivision times: Here we derive $\Pr(T_{\text{clone}} \geq L)$ for variable interdivision times. We impose the constraint that the asymptotic population growth rate (i.e. once the population reaches a stable age distribution) is equal to β, yielding a population doubling time of one unit, equal to the wild-type. Allowing an otherwise arbitrary interdivision time distribution, with probability density function $f(t)$, we thus require[16]:

$$\int_0^\infty \mathrm{e}^{-\beta t} f(t)\mathrm{d}t = \frac{1}{2} \tag{2}$$

By comparison to Equation 1, the left hand side of Equation 2 is seen to be equivalent to $\Pr(T_{\text{clone}} \geq X)$, where X is a random interdivision time. That is,

$$\Pr(T_{\text{clone}} \geq X) = \frac{1}{2} \tag{3}$$

Next, the lag time can be expressed as $L = \sum_{i=1}^{n} X_i$ where X_i are independent and identically distributed (*iid*) random variables with density $f(t)$. Then we have:

$$\begin{aligned}
\Pr(T_{\text{clone}} \geq L) &= \Pr(T_{\text{clone}} \geq \sum_{i=1}^{n} X_i) \\
&= \Pr(T_{\text{clone}} \geq X_1) \times \Pr(T_{\text{clone}} \geq \sum_{i=1}^{n} X_i \,|\, T_{\text{clone}} \geq X_1) \\
&= \Pr(T_{\text{clone}} \geq X_1) \times \Pr(T_{\text{clone}} \geq \sum_{i=2}^{n} X_i)
\end{aligned}$$

where we have used the memoryless property of the exponential distribution of T_{clone} in the last step. Continuing inductively yields:

$$\begin{aligned}\Pr(T_{\text{clone}} \geq L) &= \prod_{i=1}^{n} \Pr(T_{\text{clone}} \geq X_i) \\ &= (\Pr(T_{\text{clone}} \geq X_i))^n\end{aligned}$$

since $\{X_i\}$ are *iid*. Finally substituting Equation 3 yields:

$$\Pr(T_{\text{clone}} \geq L) = (1/2)^n = 1/c$$

2.2.2 Dominant mutations

Recall that the number of mutant clones generated in a ploidy-c population is approximately Poisson-distributed with mean $c\mu_c(N_f - N_0)$. A dominant mutation arising in a ploidy-c cell immediately yields a single phenotypic mutant; thus all mutant clones are immediately "visible". The developing time of each mutant clone is no different to a monoploid population ($T_{\text{clone}} \overset{d}{\approx} \text{Exp}(\beta)$); however, the clone size distribution is fundamentally different. In a ploidy-c population, until segregation is complete, the mutant clone contains only a single (heterozygous) phenotypic mutant descendant in each subsequent generation, according to our model of segregation (S1 Fig). After this lag of $n = \log_2 c$ generations, a purely homozygous mutant lineage arises, in which the number of phenotypic mutants doubles in each subsequent generation, as in a monoploid population.

We can readily put bounds on the range of "apparent" mutation rate, which would be estimated by the standard model, in a polyploid population. Firstly, whereas a recessive mutation yields a clone containing zero phenotypic mutants for the first n generations, a dominant mutation yields one phenotypic mutant during this segregation lag phase. Subsequently, the number of phenotypic mutants increases according to the same pattern regardless of dominance. Thus the apparent mutation rate in the dominant population must be higher than in the recessive population, which was determined above to be μ_c. Secondly, due to the segregation lag before the number of phenotypic mutants starts doubling, the number of phenotypic mutants observed in a ploidy-c population must fall short of a monoploid population with mutation rate $c\mu_c$.

Our numerical results indicated that the estimated mutation rate ($\hat{\mu}$) for a dominant mutation in a ploidy-c population does indeed typically fall between μ_c and $c\mu_c$ (S2 Fig). However, there was no constant re-scaling factor that would allow a clear interpretation of $\hat{\mu}$ in terms of the true per-copy or per-cell mutation rate. When the actual per-cell rate $c\mu_c$ was low (either

low c or low μ_c), the estimate $\hat{\mu}$ was closer to $c\mu_c$, but as $c\mu_c$ increased, $\hat{\mu}$ fell increasingly short (S2 Fig). Furthermore, the confidence intervals on the estimate became larger as ploidy increased. These results can be traced to the fundamental difference in clone size distribution: due to the segregation phase, polyploidy leads to an excess of singletons in individual clones relative to the standard model. The standard model thus provided a poor fit to the observed mutant counts, even when parameterized by $\hat{\mu}$ (S3 Fig).

The dynamics of a dominant mutation in effectively polyploid cells, along with implications for observed mutant distributions and estimated mutation rates, were actually pointed out quite early in the literature[12], but have rarely been addressed since. A more precise mathematical description of the distribution of phenotypic mutant counts for a dominant mutation in a polyploid population, and thus a basis for accurately estimating mutation rate, is a subject of our ongoing work.

2.3 Effect of single-stranded mutations

Recall that our model, like the standard model for fluctuation analysis, assumes mutations arise in double-stranded form. In reality, as explained in section 2.1 above, mutations may also arise in single-stranded form (i.e. mismatches), requiring one additional generation before producing one daughter cell with a fully fledged double-stranded mutation and one non-mutant daughter cell. To understand what difference this would make to our results, we must take into account the strand on which the mismatch arises. mRNA is transcribed from the anti-sense DNA strand. There is evidence that transcription can occur even from a mismatched heteroduplex, producing mutant mRNA and protein in the progenitor cell in which the mismatch arose[17]. As usual, we suppose that the phenotype of the cell is fully determined by its present genome content (more specifically on the anti-sense DNA strand of each chromosome copy), i.e. we neglect any additional delays in mRNA and protein turnover.

We must consider several cases depending on ploidy, dominance, and the strand on which the mismatch arises. Suppose that mismatches arise in the sense strand at per-copy rate μ_s and in the anti-sense strand at per-copy rate μ_a. For comparison to our polyploid model where mutations arise in double-stranded form, see S1 Fig.

- Cells are monoploid ($c = 1$) and the mismatch arises on the antisense strand. Then the progenitor cell is a phenotypic mutant, but there is a delay of one generation until the mutation segregates into double-stranded form in one daughter lineage, while the other

daughter lineage is wild-type. This is equivalent to our ploidy-2 model with dominant mutations arising at per-copy rate $\mu_c = \mu_a/2$.

- Cells are monoploid and the mismatch arises on the sense strand. Then the mutation will not be expressed in the progenitor, but only in the daughter lineage inheriting the double-stranded mutation. This is equivalent to our ploidy-2 model with recessive mutations arising at per-copy rate $\mu_c = \mu_s/2$.

- Cells are polyploid ($c \geq 2$) and mutations are recessive. Then regardless of the strand on which they arise, mismatches will not have a phenotypic effect on the progenitor due to masking by the other genome copies. The necessity to first segregate into double-stranded form adds one generation until achieving homozygosity and thus phenotypic expression. This is equivalent to our ploidy-$2c$ model with recessive mutations arising at per-copy rate $\mu_c = (\mu_s + \mu_a)/2$.

- Cells are polyploid, mutations are dominant and arise on the anti-sense strand. Then mismatches have immediate phenotypic effects, but there is a delay of $1 + \log_2(c) = \log_2(2c)$ generations until achieving homozygosity. This is equivalent to our ploidy-$2c$ model with dominant mutations arising at per-copy rate $\mu_c = \mu_a/2$.

- Cells are polyploid, mutations are dominant and arise on the sense strand. Then the mutant phenotype is not expressed in the progenitor, but will be expressed in the following generation in the daughter inheriting the double-stranded mutation. That is, there is a delay of one generation to produce phenotypic mutants, but $\log_2(c)$ further generations until reaching homozygosity. This is not precisely equivalent to any case of our model.

To summarize, in all but the last case, a model in which mutations initially arise as single-stranded mismatches can be converted to some form of our polyploid model in which mutations arise in double-stranded form. In reality, a mixture of mutation types will arise and contribute to the apparent mutation rate that would be estimated in a fluctuation test.

3 Effects of polyploidy on mutation rate estimation via whole-genome sequencing

Other than fluctuation tests, in recent years, bacterial mutation rates have also been estimated by whole-genome sequencing (WGS)[18;19;20;21;22]. We therefore asked whether effective poly-

ploidy also impacts WGS-based mutation rate estimates, in particular whether WGS yields the per-cell or per-copy rates. Mutation rates estimated by WGS are typically referred to as "per-genome" rates, which equal per-cell rates under the tacit assumption that bacteria are monoploid. With ploidy level taken into account, if WGS truly estimates the per-genome rates, then it has the same issue as fluctuation tests using recessive mutations. However, given that WGS-based mutation rates can be several times (up to one order of magnitude) higher than rates from fluctuation tests[18;19;20], it is also plausible that WGS actually yields per-cell rates that reflect the underlying ploidy level. To our knowledge, however, the impact of polyploidy on WGS-based mutation rates has never been explored.

To date, there are three experimental methods used to estimate mutation rates in combination with WGS. They are mutation accumulation (MA)[21], counting neutral mutations fixed such as in the *E. coli* long-term evolutionary experiment (LTEE)[18], and maximum-depth sequencing (MDS)[19]. These three WGS methods yield per-genome or per-nucleotide mutation rates within the same order of magnitude of one another, for both *E. coli* and *Salmonella*[18;19]. MA is the most commonly used method[20;21;22]. In MA experiments, bacteria accumulate mutations by serial-passaging on solid media with single-colony bottlenecks to minimize the impact of selection[21]. For fast-growing bacteria like *E. coli*, forming a colony requires 25-30 divisions and can occur in one day[20;21]. The daily passaging continues until a high number of generations, such as 6000 (ref.[20]). This is also routinely done in replicate cultures to reach a high total generation number such as 133,476 (ref.[20]). In the end, one bacterial clone, after forming one final colony, is sequenced. Base-substitution mutations or SNPs are typically called with a high threshold such as >80% or even 100% (ref.[18;20]). Within-colony polymorphisms are thereby ignored. This implies that only homozygous mutant alleles in the cell that founded the final colony are registered in WGS. If the total generation number is high enough, the last 25-30 generations will have a negligible impact on the mutation rate estimate. Since MA remains the most widely used method, we focus on it to decipher how effective polyploidy impacts WGS-based mutation rate estimation.

In the MA assay, as described above, a single colony (assumed to be founded by a single cell) is picked at each bottleneck to continue propagation, and at the final time point for sequencing. Thus, one cell is chosen, presumably uniformly at random, among all existing cells in the population at each sampling time. In order to quantify the mutations accumulated along its direct lineage, we would need to follow this lineage backwards in time to the common ancestor that founded the population. In general, a typical "backward lineage" does not show the same

statistical properties – particularly the number of cell divisions elapsed – as a typical "forward lineage"[23]. However, if we make the important simplifying assumptions that interdivision time is fixed (in particular, not affected by any mutations) and there is no cell death, then it is equivalent to choose a lineage at random either backwards or forwards in time. Thus, we simplify the process by considering cell divisions forwards in time along a single focal lineage throughout the entire experiment, from the founder cell to the cell forming the colony picked for sequencing. At each cell division, we thus randomly choose one daughter cell to be the ancestor of the chosen cell, i.e. to remain in the sampled lineage, and "discard" the other daughter cell from consideration (S4 Fig, A).

In this model, the probability of a cell acquiring a mutation on any of its genome copies within one generation is given by $c\mu_g$, with μ_g the per-genome-copy mutation rate and c the effective ploidy. For a mutation to be registered in WGS, it must fix in the sampled lineage. If cells are monoploid, then the mutant progenitor and its offspring are all homozygous, and all mutations occurring in the sampled lineage will therefore be passed down the lineage and registered in WGS. For a polyploid cell that acquires one mutation, however, only a fraction $1/c$ of its descendants will become homozygous mutants while the rest become genetically wild-type, due to asymmetric inheritance that we showed experimentally (main text Fig 3). To register a mutation requires that one picks up a homozygous mutant descendant of the mutant progenitor (S4 Fig, B-C), which occurs with probability $1/c$. Therefore, although polyploidy increases the influx of mutations by a factor c, this is cancelled out by a decrease of factor c in the probability that a mutation fixes in the sampled lineage, leaving μ_g as the apparent mutation rate. This result may not be exact if cell division times are variable, since our model of sampling one cell at random at each cell division forwards in time is then not precisely equivalent to sampling at random from all cells existing at a given time point in the real MA assay. However, the argument shows that we will not in general recover the per-cell mutation rate, and instead expect something close to the per-genome-copy mutation rate.

In the end, then, mutation rate estimates derived by WGS applied to MA assays represent (approximately) per-genome-copy rates, comparable to fluctuation tests with recessive mutations returning per-target-copy rates. Effective polyploidy thus does not explain the aforementioned discrepancy of up to an order of magnitude between WGS-based and fluctuation test-based estimates of mutation rate. Although both methods are impacted similarly by effective polyploidy, the mechanisms are fundamentally different. In fluctuation tests, effective polyploidy introduces phenotypic delay and masks heterozygous mutants from selection. In mu-

tation accumulation, mutations are assumed to be neutral, but asymmetric inheritance caused by effective polyploidy decreases their fixation probability.

Although we focused on MA, both of the two aforementioned alternative WGS methods, LTEE- and MDS-based mutation rates, yield mutation rate estimates of the same order of magnitude as MA-based rates[18;19]. In LTEE, counting neutral mutations actually yields slightly lower mutation rates than MA and MDS[18]. In MDS, since mutation rates are estimated based on polymorphism in whole-population samples[19], the resulting per-nucleotide mutation rates are literally per-nucleotide, which translates readily to per-genome rates for any given genome size. However, as we have shown here, these estimated rates must be interpreted carefully as they do not reflect the per-cell mutation rate.

4 Standing genetic variation and rescue

4.1 General model notes

In this section we develop the models we use to investigate the evolutionary consequences of effective polyploidy (main text Fig 7). Recall that in our framework, every cell contains c $(= 1, 2, 4, 8, \ldots)$ copies of the locus of interest, which for brevity we refer to as "ploidy", though chromosomes may only be partial. At each generation these chromosomes double and are evenly divided between the two daughter cells, splitting at the deepest point in their genealogy. A cell's type is defined by the number of mutant chromosomes (out of c) that it carries: type $j = 0$ is homozygous wild-type; types $j = 2^i$, $i = 0, 1, \ldots, n-1$ are heterozygous; type $j = 2^n = c$ is homozygous mutant.

We model population dynamics in discrete generations. To capture fitness differences for bacteria undergoing binary fission, we suppose a type j cell successfully divides before dying, i.e. produces two offspring rather than zero, with probability p_j. These probabilities will differ between the old environment (to calculate mutation-selection balance) and the new environment (to calculate the probability of rescue). In the deterministic calculation of the mutation-selection balance below, only the proportion of offspring of each type matters; thus, the results are not actually particular to organisms reproducing by binary fission, but do depend on the types of offspring that can be produced, determined here by the assumed pattern of chromosome segregation. In the stochastic model for rescue, the entire probabilistic distribution of offspring numbers matters, not only expected values.

Consistent with the population genetics literature, here we define mutation rate (per cell, per generation) as the proportion of offspring that are mutant (in a deterministic model) or the probability that each offspring independently is mutant (in a stochastic model). Again, we suppose mutations arise in double-stranded form (cf. discussion in sections 2.1 and 2.3 above). For ploidy c, a wild-type cell has mutation rate $\tilde{\mu} = c\tilde{\mu}_c = 2^n\tilde{\mu}_c$ where $\tilde{\mu}_c$ is the mutation rate per copy. Although it is possible that more than one copy mutates simultaneously in this model, these second-order events will drop out of the approximations we derive.

We choose our notation ($\tilde{\mu}$) to make a subtle distinction from the mutation rate parameter (μ) used in the fluctuation test model (section 2). Recall that the standard definition of mutation rate in the fluctuation analysis literature is the number of mutants produced per wild-type cell per division; that is, a mutation event, occurring with probability μ, retains one wild-type cell and produces one new mutant[6]. In contrast, in the population genetics approach, each offspring has a probability $\tilde{\mu}$ to become a mutant. Assuming two offspring as in binary fission, the probability of producing one mutant upon wild-type cell division is thus, to first order, $2\tilde{\mu}$. Therefore, a bacterial mutation rate estimated by standard methods from a fluctuation test should be divided by two in order to parameterize a typical population genetics model. This discrepancy in the interpretation of mutation rate has not, to our knowledge, been explicitly recognized, as such models are rarely treated side by side. In order to maintain consistency with each existing body of literature, such that we recover standard results in the monoploid case, we use the two different definitions of mutation rate in our respective modeling approaches.

4.2 Mutation-selection balance (deterministic)

Here we suppose mutations are costly (where this cost may be masked in heterozygotes, depending on the dominance assumption) and calculate the frequency of the mutant types at equilibrium.

4.2.1 Mathematical derivations

Let $x_j(t)$ denote the frequency (proportion) of type j in the population at generation t, and w_j the relative fitness of type j, where fitness is given by the expected number of surviving offspring, and relative fitnesses are normalized by the wild-type. (Under our binary fission model where individuals have two offspring with probability p_j or zero offspring with probability $1 - p_j$, we

have $w_j = p_j/p_0$.) We denote population mean fitness by $\bar{w}(t) := \sum_j w_j x_j(t)$. A proportion $\tilde{\mu}$ of type 0 individuals' offspring mutate to type 1, while the rest remain type 0. According to our model of chromosome segregation (see main text Materials and Methods), type 2^i individuals ($0 \le i \le n-1$) produce half type 0 and half type 2^{i+1} offspring, while type $c = 2^n$ individuals produce only type 2^n offspring. Then for $c > 1$ the type frequencies can be described by the following recursions (with census after mutation and before selection):

$$x_0(t+1) = \frac{(1-\tilde{\mu})w_0 x_0(t) + \frac{1}{2}\sum_{i=0}^{n-1} w_{2^i} x_{2^i}(t)}{\bar{w}(t)} \tag{4a}$$

$$x_1(t+1) = \frac{\tilde{\mu} w_0 x_0(t)}{\bar{w}(t)} \tag{4b}$$

$$x_{2^i}(t+1) = \frac{\frac{1}{2} w_{2^{i-1}} x_{2^{i-1}}(t)}{\bar{w}(t)}, \quad \text{for } 1 \le i \le n-1 \tag{4c}$$

$$x_{2^n}(t+1) = \frac{\frac{1}{2} w_{2^{n-1}} x_{2^{n-1}}(t) + w_{2^n} x_{2^n}(t)}{\bar{w}(t)} \tag{4d}$$

Provided the mutation rate is small compared to fitness costs, mutants are rare and the wild-type dominates the population: $x_0(t) \approx 1$ and $\bar{w}(t) \approx 1$. Then the equilibrium frequencies, x_j^*, approximately satisfy:

$$x_1^* \approx \tilde{\mu} \tag{5a}$$

$$x_{2^i}^* \approx \frac{w_{2^{i-1}} x_{2^{i-1}}^*}{2}, \quad \text{for } 1 \le i \le n-1 \tag{5b}$$

$$x_{2^n}^* \approx \frac{w_{2^{n-1}} x_{2^{n-1}}^*}{2} + w_{2^n} x_{2^n}^* \tag{5c}$$

This yields the general solution for the mutation-selection balance in polyploids under our model of chromosome segregation:

$$x_1^* \approx \tilde{\mu} = 2^n \tilde{\mu}_c \tag{6a}$$

$$x_{2^i}^* \approx \left(\prod_{j=0}^{i-1} w_{2^j}\right) \frac{\tilde{\mu}}{2^i} = \left(\prod_{j=0}^{i-1} w_{2^j}\right) 2^{n-i} \tilde{\mu}_c, \quad \text{for } 1 \le i \le n-1 \tag{6b}$$

$$x_{2^n}^* \approx \frac{\prod_{j=0}^{n-1} w_{2^j}}{1 - w_{2^n}} \cdot \frac{\tilde{\mu}}{2^n} = \frac{\prod_{j=0}^{n-1} w_{2^j}}{1 - w_{2^n}} \tilde{\mu}_c \tag{6c}$$

We assume the mutation has fitness cost s in a homozygous mutant relative to a homozygous wild-type; that is, $w_{2^n} = 1 - s$. The condition that mutants are rare amounts to $\tilde{\mu} \ll s \le 1$. We can then consider various dominance models to determine the fitness of the heterozygotes.

When the mutation is completely recessive, only homozygous mutants bear the cost, while heterozygotes have the same fitness as wild-types: $w_{2^i} = w_0 = 1$ for $0 \leq i \leq n-1$. Substituting into the general solution yields:

$$x^*_{2^i} \approx \frac{\tilde{\mu}}{2^i} = 2^{n-i}\tilde{\mu}_c, \quad \text{for } 0 \leq i \leq n-1 \text{ (heterozyg.)} \tag{7a}$$

$$x^*_{2^n} \approx \frac{\tilde{\mu}}{2^n s} = \frac{\tilde{\mu}_c}{s} \quad \text{(homozyg.)} \tag{7b}$$

Then the total frequency of the mutant allele, i.e. the proportion of all alleles in the population that are mutant, is:

$$\sum_{i=0}^{n} \frac{2^i}{2^n} x^*_{2^i} \approx (n-1)\tilde{\mu}_c + \frac{\tilde{\mu}_c}{s} \tag{8}$$

When the mutation is completely dominant, all heterozygotes have the same fitness as homozygous mutants: $w_{2^i} = 1 - s$ for $0 \leq i \leq n$. Substituting into the general solution yields:

$$x^*_{2^i} \approx \left(\frac{1-s}{2}\right)^i \tilde{\mu} = (1-s)^i 2^{n-i}\tilde{\mu}_c, \quad \text{for } 0 \leq i \leq n-1 \text{ (heterozyg.)} \tag{9a}$$

$$x^*_{2^n} \approx \left(\frac{1-s}{2}\right)^n \frac{\tilde{\mu}}{s} = (1-s)^n \frac{\tilde{\mu}_c}{s} \quad \text{(homozyg.)} \tag{9b}$$

The total mutant allele frequency is then:

$$\sum_{i=0}^{n} \frac{2^i}{2^n} x^*_{2^i} \approx \frac{\tilde{\mu}_c}{s} \tag{10}$$

For comparison, taking the same solution approach for monoploids ($c = 1$ and $w_1 = 1 - s$) recovers the standard result for mutant frequency,

$$x^*_1 \approx \frac{\tilde{\mu}}{s} \equiv \frac{\tilde{\mu}_c}{s} \tag{11}$$

4.2.2 Results

The key expressions in the cases of complete dominance or complete recessivity (Equations 7-11) are summarized in Table 1 in the main text, and an example of mutant allele frequency as a function of cost is plotted in S5 Fig.

In the completely recessive case, the cost is masked in heterozygotes, and thus the mutant allele is maintained at a higher overall frequency than in a monoploid population. Meanwhile, the frequency of homozygous mutants is independent of ploidy.

In the completely dominant case, both heterozygotes and homozygotes are subject to selection and thus present at a lower frequency than in the recessive case. The total mutant

allele frequency turns out to be exactly the same as in the monoploid case. However, since heterozygote cells contribute less than 100% mutant chromosomes, the frequency of cells containing at least one mutant chromosome must necessarily be larger than in the monoploid case, where every mutant cell is weighted by a 100% contribution. Indeed, the total frequency of heterozygous and homozygous mutants among all cells is given by:

$$\sum_{i=0}^{n} x_{2^i}^* \approx \frac{\tilde{\mu}_c}{s}\left(\frac{2^{n+1}s + (1-s)^{n+1}}{1+s}\right) \tag{12}$$

The factor in brackets can be shown to be larger than one for any $s \in (0, 1]$ and $n \geq 1$, and increasing with n. That is, the total frequency of hetero- and homozygous mutants increases with ploidy ($c = 2^n$). However, a c-fold increase in ploidy yields less than a c-fold increase in this total mutant cell frequency. This is because mutational influx is proportional to c, but half of heterozygous mutants' offspring are wild-type and thus "lost" from this pool.

For comparison, Otto and Whitton[24] gave the equilibrium frequency of a costly mutant allele to be $\tilde{\mu}_c/\sigma_c$, where σ_c is "the fitness effects of a single mutation in an organism of ploidy level c" (p. 417, ref.[24]), allowing any level of dominance except the completely recessive case. This result is given for monoploids, diploids, or tetraploids, for either asexual or randomly mating sexual reproduction (with particular assumptions on chromosome segregation in tetraploids). Our result, for asexual reproduction with any ploidy of the form $c = 2^n$, is thus consistent in the special case of complete dominance. Furthermore, these authors found that the mutant allele frequency "increases with ploidy level for deleterious mutations that are partially recessive and masked" (p. 417, ref.[24]). Our result in the limiting case of completely recessive mutations is consistent with this trend.

4.3 Rescue (stochastic)

Suppose a population is exposed to a harsh new environment (e.g. antibiotics), such that the basic reproductive number (R_0) of phenotypically wild-type ("sensitive") cells does not exceed one, and thus the population will decline to extinction unless it is rescued by phenotypically mutant ("resistant") cells with R_0 larger than one. We are interested in the probability that the population is rescued, due to the establishment of at least one resistant lineage, from the standing genetic variation (mutant types that already arose in the old environment) and/or *de novo* mutations that occur in the new environment.

4.3.1 Mathematical derivations

Recall that in our framework, a type j cell has two daughters with probability p_j, and otherwise dies without leaving offspring. Thus $R_{0,j} = 2p_j$ and the rescue scenario amounts to taking $p_j = p_S \leq 1/2$ for all types j that are sensitive in the new environment, and $p_j = p_R > 1/2$ for all types j that are resistant. (Recall that $j = 0$ corresponds to the homozygous wild-type; $j = 2^i$ for $0 \leq i \leq n-1$ correspond to heterozygotes; and $j = 2^n = c$ corresponds to the homzygous mutant.) We now model population dynamics stochastically using a multi-type branching process.

The offspring distributions of the various types are described by the following probability generating functions, where $\vec{z} = (z_0, z_1, \ldots, z_{2^n})$ is a dummy variable:

$$f_0(\vec{z}) = 1 - p_0 + p_0\left((1-\tilde{\mu})z_0 + \tilde{\mu}z_1\right)^2 \tag{13a}$$

$$f_{2^i}(\vec{z}) = 1 - p_{2^i} + p_{2^i} z_0 z_{2^{i+1}}, \qquad \text{for } 0 \leq i \leq n-1 \tag{13b}$$

$$f_{2^n}(\vec{z}) = 1 - p_{2^n} + p_{2^n} z_{2^n}^2 \tag{13c}$$

The extinction probabilities ζ_j starting from a single type j are given by the smallest non-negative fixed point[25], $\vec{f}(\vec{\zeta}) = \vec{\zeta}$. That is,

$$\zeta_0 = 1 - p_0 + p_0\left((1-\tilde{\mu})\zeta_0 + \tilde{\mu}\zeta_1\right)^2 \tag{14a}$$

$$\zeta_{2^i} = 1 - p_{2^i} + p_{2^i}\zeta_0\zeta_{2^{i+1}}, \qquad \text{for } 0 \leq i \leq n-1 \tag{14b}$$

$$\zeta_{2^n} = 1 - p_{2^n} + p_{2^n}\zeta_{2^n}^2 \tag{14c}$$

The equation for ζ_{2^n} is decoupled and can readily be solved for the two roots, $\zeta_{2^n} = 1$ and $\zeta_{2^n} = 1/p_{2^n} - 1$. The former solution gives the extinction probability when $p_{2^n} \leq 1/2$, while the latter applies when $p_{2^n} > 1/2$, which is the case of interest in the rescue scenario. Thus,

$$\zeta_{2^n} = 1/p_R - 1 \tag{15}$$

Next, working backwards from $i = n-1$ to $i = 0$ to successively substitute the expression for $\zeta_{2^{i+1}}$ into the equation for ζ_{2^i}, we arrive at the general form,

$$\zeta_{2^i} = 1 - p_{2^i} + \sum_{k=i+1}^{n-1}\left(\prod_{\ell=i}^{k-1} p_{2^\ell}\right)(1 - p_{2^k})\zeta_0^k + \left(\prod_{\ell=i}^{n-1} p_{2^\ell}\right)\zeta_0^{n-i}\zeta_{2^n}, \qquad \text{for } 0 \leq i \leq n-1 \tag{16}$$

The terms in this sum reflect all possible paths to extinction, whereby either a cell in the mutant lineage fails to divide at some step before complete segregation, or cell divisions are successful

up to complete segregation but the homozygous mutant fails to establish a surviving lineage, while all homozygous wild-type descendants (one produced per cell division until complete segregation) also lead to extinction.

These equations could in general be solved numerically. For $\tilde{\mu} \ll 1$, we obtain the analytical approximation:

$$\zeta_{2^i} = 1 - p_{2^i} + \sum_{k=1}^{n-i-1} (1 - p_{2^{i+k}}) \left(\prod_{\ell=i}^{i+k-1} p_{2^\ell} \right) + \left(\prod_{\ell=i}^{n-1} p_{2^\ell} \right) \zeta_{2^n} + \mathcal{O}(\tilde{\mu}), \quad \text{for } 0 \le i \le n-1 \tag{17a}$$

$$\zeta_0 = 1 - \frac{2(1 - \tilde{\zeta}_1)}{1/p_0 - 2} \tilde{\mu} + \mathcal{O}(\tilde{\mu}^2) \tag{17b}$$

where $\tilde{\zeta}_{2^i}$ denotes the zero-order term in ζ_{2^i} as given in (17a). The approximation for ζ_{2^i} essentially neglects the chance that another mutation occurs and escapes extinction among the wild-type descendants produced before complete segregation. The approximation for ζ_0 (and thus also for all types 2^i) will break down as $p_0 \to 1/2$, i.e. the type 0 branching process approaches criticality. On the other hand, the special case $p_0 = 0$ leads to the exact solutions $\zeta_0 = 1$ and $\zeta_{2^i} = \tilde{\zeta}_{2^i}$.

In the special case of complete recessivity or dominance, all heterozygotes have the same division probability, $p_{2^i} = p_{\text{het}}$ for $0 \le i \le n-1$, where $p_{\text{het}} = p_S$ for recessivity or $p_{\text{het}} = p_R$ for dominance. Substituting Equation 15 for ζ_{2^n}, the expressions for ζ_{2^i} can then further be simplified to the exact form:

$$\zeta_{2^i} = (1 - p_{\text{het}}) \frac{1 - (p_{\text{het}}\zeta_0)^{n-i}}{1 - p_{\text{het}}\zeta_0} + (p_{\text{het}}\zeta_0)^{n-i}(1/p_R - 1), \quad \text{for } 0 \le i \le n-1 \tag{18}$$

or, for $\tilde{\mu} \ll 1$, the approximate form:

$$\zeta_{2^i} = 1 - p_{\text{het}}^{n-i}(2 - 1/p_R) + \mathcal{O}(\tilde{\mu}), \quad \text{for } 0 \le i \le n-1 \tag{19a}$$

$$\zeta_0 = 1 - 2p_{\text{het}}^n \frac{2 - 1/p_R}{1/p_S - 2} \tilde{\mu} + \mathcal{O}(\tilde{\mu}^2) \tag{19b}$$

Rescue from standing genetic variation: We first consider the contribution to rescue from mutant types (both hetero- and homozygous) that are already present when the environment switches. For this purpose, we neglect *de novo* mutations in the new environment, thus setting $\tilde{\mu} = 0$ in the solutions for the extinction probabilities ζ_j. The probability of rescue from standing genetic variation, denoted P_{SGV}, is calculated as the the complement of the probability that all

starting types ultimately fail to establish surviving lineages, that is:

$$P_{\text{SGV}} = 1 - \prod_{i=0}^{n} \zeta_{2^i}^{N_{2^i}} = 1 - \exp\left(\sum_{i=0}^{n} N_{2^i} \log(\zeta_{2^i})\right) \tag{20}$$

where N_j is the number of type j individuals present at the time the environment switches. If we assume the population begins at the deterministic mutation-selection balance calculated in the previous section, with total population size N independent of ploidy (see ref.[24] for a discussion of possible links that we neglect), then $N_j = N x_j^*$ where $x_j^* \propto \tilde{\mu}_c$, with the proportionality constant depending on dominance in the old environment and on ploidy (main text Table 1). The extinction probabilities ζ_{2^i} are given in general by Equation 17a, or in the case of complete dominance or recessivity in the new environment by Equation 19a, setting $\tilde{\mu} = 0$.

If we assume both the cost in the old environment and the resistant phenotype in the new environment are completely recessive, we substitute Equation 7 for the approximation of $x_{2^i}^*$ and Equation 19a (with $p_{\text{het}} = p_S$ and $\tilde{\mu} = 0$) for ζ_{2^i} to obtain:

$$P_{\text{SGV,R/R}} \approx 1 - \exp\left(m \cdot \left[\sum_{i=0}^{n-1} 2^{n-i} \log(1 - p_S^{n-i}(2 - 1/p_R)) + \log(1/p_R - 1)/s\right]\right) \tag{21}$$

where $m := N\tilde{\mu}_c$ is a ploidy-independent measure of mutational influx in the population, given by the product of initial population size and per-copy mutation rate. In the case of complete dominance in both environments, we substitute Equation 9 for the approximation of $x_{2^i}^*$ and Equation 19a (with $p_{\text{het}} = p_R$ and $\tilde{\mu} = 0$) for ζ_{2^i} to obtain:

$$P_{\text{SGV,D/D}} \approx 1 - \exp\left(m \cdot \left[\sum_{i=0}^{n-1} 2^{n-i}(1-s)^i \log(1 - p_R^{n-i}(2 - 1/p_R)) + \log(1/p_R - 1)(1-s)^n/s\right]\right) \tag{22}$$

A caveat is that we have taken all types to be at their deterministic frequencies. This assumption will tend to be unrealistic when mutational influx (m) is small. Stochastic effects imply that mutant cell types could be absent all together, and the realized frequencies of various types will be correlated through time. In particular, rescue could fail even if $p_R = 1$ due to the chance that resistant cells are absent at any given time, whereas our current approach yields $P_{\text{SGV}} = 1$ when $p_R = 1$, since the average frequency of resistant cells is non-zero. In general, stochasticity in realized mutant frequencies is expected to reduce P_{SGV}[26]. Nonetheless, we do not expect changes to the general qualitative effects of ploidy and other model parameters on rescue probability. We thus leave a stochastic treatment of the SGV for future theoretical work.

Rescue from *de novo* mutations: We now consider the contribution to rescue from mutations that arise in the new environment. For this purpose, we suppose the population is initially composed of N homozygous wild-type (type 0) individuals. The probability of rescue from *de novo* mutations, denoted P_{DN}, is then calculated as:

$$P_{\text{DN}} = 1 - \zeta_0^N \tag{23}$$

Clearly, P_{DN} can only be non-zero when $p_S > 0$, such that existing wild-type cells can produce offspring. Assuming that $\tilde{\mu} \ll 1$ (such that $1 - \zeta_0 \ll 1$) and that N is large, we can approximate[27]:

$$P_{\text{DN}} \approx 1 - \mathrm{e}^{-N(1-\zeta_0)} \approx 1 - \exp\left(-2^n m\alpha\right) \tag{24}$$

where α is the coefficient of $\tilde{\mu}$ in the approximation of ζ_0 (Equation 17b or 19b), and again $m := N\tilde{\mu}_c$. In particular, when the mutation is completely recessive in the new environment, we have

$$P_{\text{DN,R}} \approx 1 - \exp\left(-2^n \cdot m \cdot 2p_S^n \frac{2 - 1/p_R}{1/p_S - 2}\right) \tag{25}$$

while when the mutation is completely dominant in the new environment, we have

$$P_{\text{DN,D}} \approx 1 - \exp\left(-2^n \cdot m \cdot 2p_R^n \frac{2 - 1/p_R}{1/p_S - 2}\right) \tag{26}$$

4.3.2 Results

Here we explore the effects of model parameters, ploidy, and dominance on the probability of rescue. We assume throughout that the mutation rate is small ($\tilde{\mu} = c\tilde{\mu}_c \ll 1$ for reasonable c), allowing us to use analytical approximations derived above. For simplicity we will only illustrate the cases of complete recessivity or dominance. Furthermore, in the case of SGV, we suppose here that dominance is the same in both the old and new environments, although there is empirical evidence that dominance can in fact be environment-dependent[28] and this situation could also readily be considered using our general mathematical results.

With these simplifying assumptions, we apply Equations 21 and 22 to calculate P_{SGV}, and Equations 25 and 26 to calculate P_{DN}. Recall that P_{SGV} covers the contribution of all hetero- and homozygous mutant types existing at the time of the environmental shift, while P_{DN} covers the contribution of *de novo* mutations arising in the new environment from homozygous wild-type cells. We also consider the overall probability of rescue from either (not mutually exclusive)

source:

$$
\begin{aligned}
P_{\text{tot}} &= 1 - (1 - P_{\text{SGV}})(1 - P_{\text{DN}}) \\
&= P_{\text{SGV}} + P_{\text{DN}} - P_{\text{SGV}} P_{\text{DN}}
\end{aligned} \tag{27}
$$

The plots in S6 Fig – S9 Fig illustrate the effects of model parameters on the aforementioned probabilities (P_{SGV}, P_{DN}, P_{tot}) as well as the ratio $P_{\text{SGV}}/P_{\text{DN}}$, for various ploidy levels.

Effect of probabilities that sensitive and resistant cells divide before death in the new environment (p_S, p_R): P_{SGV} is independent of p_S in the dominant case, since both heterozygous and homozygous mutant types are resistant. In the recessive case, since heterozygotes are sensitive, P_{SGV} is increasing with p_S. In the extreme when $p_S = 0$, rescue can only occur from pre-existing homozygous mutants, whose frequency is independent of ploidy (main text Table 1). P_{DN} increases with p_S in both the recessive and dominant cases, since increasing the chance that wild-type cells produce surviving offspring increases the opportunities for *de novo* mutations. However, P_{DN} is more sensitive to p_S in the recessive case, since changes in p_S affect survival of heterozygotes as well as homozygotes. Taken together, P_{tot} is clearly increasing with p_S. Furthermore, P_{SGV}, P_{DN} and P_{tot} are all increasing with p_R, by reducing the chance that a resistant lineage goes extinct. The relative contribution of SGV ($P_{\text{SGV}}/P_{\text{DN}}$) is greatest at low p_S, where wild-type cells are unlikely to survive to produce new mutants after the environment changes, with this dependency stronger in the recessive case. The dependency on p_R is weak and not in a consistent direction.

Effect of mutational influx ($m = N\tilde{\mu}_c$): The larger the population (N) or the higher the per-copy mutation rate ($\tilde{\mu}_c$), the greater the number of mutant cells in the SGV and the more likely new mutations are to be generated. Thus, P_{SGV}, P_{DN} and P_{tot} are all increasing with $m = N\tilde{\mu}_c$. The relative contribution of SGV ($P_{\text{SGV}}/P_{\text{DN}}$) is greatest at low mutational influx.

Effect of fitness cost in the old environment (s): The higher the cost of the mutation, the lower its frequency in the old environment; thus P_{SGV} is decreasing with s. Mutations that first arise in the new environment do not incur this cost (our model takes fitness in the new environment, determined by p_R, independent of fitness in the old environment, determined by s) and thus P_{DN} is independent of s. Therefore, both the overall probability of rescue (P_{tot}) and the relative contribution of SGV ($P_{\text{SGV}}/P_{\text{DN}}$) are decreasing with s.

Effect of ploidy ($c = 2^n$): As described in the main text, P_{SGV} increases with ploidy in the recessive case (for $p_S > 0$) and in the dominant case. Meanwhile, P_{DN} decreases with ploidy in the recessive case and increases in the dominant case. These patterns are robust to variations in parameter values (S6 Fig – S9 Fig).

Our results for rescue from standing genetic variation, if we assume a deterministic mutation-selection balance prior to environmental change, interestingly qualitatively agree with and extend our results for fluctuation analysis, which models the stochastic accumulation of genetic variation in an exponentially growing population with mutations assumed to be cost-free. In a fluctuation test, obtaining mutant colonies growing on selective plates corresponds to rescue, with the assumption that every phenotypic mutant produces a visible colony ($p_R = 1$) while every phenotypically wild-type cell fails to produce a visible colony ($p_S = 0$).

In the recessive case, both the deterministic frequency of homozygous mutants at mutation-selection balance (section 4.2.2) and the distribution of number of homozygous mutants in an exponentially growing culture (section 2.2) turn out to be independent of ploidy. If $p_S = 0$, as in fluctuation analysis and a special case of our rescue model, the observed mutant distribution in the former or $P_{\text{SGV,R/R}}$ in the latter thus reflect only the contribution of homozygous mutants that are immediately resistant in the new environment. Thus the result is unaffected by ploidy. As p_S increases in our more general rescue model, heterozygous mutants can also contribute to rescue. Their frequency in the SGV (main text Table 1), and thus $P_{\text{SGV,R/R}}$, increases with ploidy. However, the increase in rescue probability is tempered by heterozygotes' lower probability of escaping extinction, due to the higher chance of loss until complete segregation has occurred.

In the dominant case, both hetero- and homozygous mutant cells are phenotypically mutant, i.e. immediately resistant in the new environment. Their total frequency in the SGV at mutation-selection balance (section 4.2.2), similarly to a fluctuation test (section 2.2), increases with ploidy, but falls short of the pattern expected for c-times higher mutation rate in the standard monoploid model. This is explained by the segregation lag in heterozygous mutants, during which only half of offspring are likewise phenotypic mutants. For the same reason, the probability that a lineage initiated by a heterozygous mutant escapes extinction in the new environment is lower than that of a homozygous mutant (whenever $p_R < 1$), tempering their contribution to rescue.

Our results for rescue from *de novo* mutations also reflect the counteracting effects of higher mutational influx but a longer segregation lag as ploidy increases. In the case of a completely

recessive mutation, the net effect is a reduction of P_{DN} with ploidy. Consider the expression from Equation 25:

$$P_{\text{DN,R}} \approx 1 - \exp\left(-2^n \cdot m \cdot 2p_S^n \frac{2 - 1/p_R}{1 - p_S/2}\right)$$

The factor 2^n reflects the gain in mutational influx with ploidy, while the factor p_S^n reflects the number of successful divisions of sensitive cells required for complete segregation of mutant chromosomes. Since $p_S < 1/2$ by assumption, the net effect is that $(2p_S)^n$ and in turn $P_{\text{DN,R}}$ are decreasing with n. Intuitively, each doubling of ploidy doubles the mutational influx, but requires one additional cell division, occurring with probability less than one half, to produce a resistant cell.

On the other hand, for a completely dominant mutation, the net effect is an increase of P_{DN} with ploidy. Consider Equation 26:

$$P_{\text{DN,D}} \approx 1 - \exp\left(-2^n \cdot m \cdot 2p_R^n \frac{2 - 1/p_R}{1 - p_S/2}\right)$$

The detrimental effect of the segregation lag even on dominant mutants is reflected by p_R^n, which decreases with n (unless $p_R = 1$). However, this is now more than compensated by the gain in mutational influx (factor 2^n). By assumption, $p_R > 1/2$, thus $(2p_R)^n$ and in turn $P_{\text{DN,D}}$ increase with n.

Putting together the effects of ploidy on both SGV and *de novo* sources, we find that the overall probability of rescue, P_{tot}, depends only weakly on ploidy in the recessive case, but is increasingly contributed by SGV at higher ploidy. In the dominant case, P_{tot} is clearly increasing with ploidy. The relative contribution of SGV ($P_{\text{SGV}}/P_{\text{DN}}$) tends to be decreasing over reasonable ploidy levels, but this trend can reverse at higher ploidy, especially when s and/or p_S are large. We briefly note that if we were to account for stochasticity in the number of mutants in the SGV, just as we already accounted for stochasticity in the number of *de novo* mutations occurring, we would expect the contribution of SGV to decrease (see derivation of P_{SGV} above).

It is interesting to compare our results on rescue to previous results on the rate of substitutions or adaptation in a constant-sized population. As a baseline, in the case of neutral mutations, any individual chromosome has equal probability of ultimately fixing in the population. In a population of N individuals of ploidy c, where each chromosome mutates at rate μ_c, the overall substitution rate is thus (mutation influx rate) $\times$ (fixation probability) $= (c\mu_c N) \times 1/(cN) = \mu_c$, which is independent of ploidy, as in the mutation accumulation assay (section 3). On the other hand, selection either against deleterious mutations (as examined

in the mutation-selection balance, section 4.2) or for beneficial mutations (as examined here) introduces dependencies on ploidy. Previously, Otto and Whitton[24] considered how the "rate of adaptation", defined as the rate of fitness increase due to adaptive substitutions, depends on ploidy. For adaptation from a single, previously deleterious allele in the SGV that becomes beneficial, they found that the rate of adaptation increases with ploidy if the selective benefit of the allele when in a single copy is sufficiently large relative to the prior selective disadvantage, and the population size is not too large. In the rescue situation, the population is declining and mutations are strongly favoured. Thus this previous result is in qualitative agreement with our results for rescue, where P_{SGV} increases with ploidy for both recessive and dominant mutations. For adaptation from ongoing, beneficial *de novo* mutations, in an asexual population, Otto and Whitton found that adaptation rate in a small population increases with ploidy for sufficiently dominant mutations, but decreases with ploidy for more recessive mutations. Again, this is in qualitative agreement with our finding that $P_{\mathrm{DN,D}}$ increases but $P_{\mathrm{DN,R}}$ decreases with ploidy.

Effect of dominance: P_{DN} is always larger for the dominant case than for the recessive case, since in the former, heterozygotes are already capable of rescuing the population, whereas in the latter, only homozygous mutants can rescue. The idea that recessive adaptive mutations are at a disadvantage in establishment dates back to Haldane[29] and is now commonly known as "Haldane's Sieve"[30]. Moreover, in our model, P_{DN} increases with ploidy for dominant mutations and decreases for recessive mutations; thus the advantage of dominance clearly is greater at higher ploidy. These effects of ploidy, and thus the magnitude of the difference between recessive and dominant mutations, are weakest at high p_S (as this increases the chance that recessive mutations survive until homozygosity) and low p_R (as this reduces the advantage of dominant mutations being expressed in heterozygotes).

In contrast, the effect of dominance on P_{SGV} can go in either direction. Indeed, Orr and Betancourt[30] have previously pointed out that Haldane's Sieve does not hold for adaptation from standing genetic variation, since more dominant mutations have higher per-copy probability of establishment but are present at lower frequency. Specifically, in our model $P_{\mathrm{SGV,R/R}}$ can exceed $P_{\mathrm{SGV,D/D}}$ when p_S is sufficiently high and p_R sufficiently low (for the same reasons that the advantage of dominance weakens in P_{DN}), or when s is high (so that masking this cost in heterozygotes yields a greater advantage to recessive mutations). Our results are in qualitative agreement with Orr and Betancourt[30], who found (with the implicit assumption that dominance coefficients are not environment-dependent) that completely recessive mutations

have a higher probability of establishment than (semi-)dominant mutations when the selective disadvantage in the old environment is high and/or the benefit in the new environment is small.

References

[1] Sharan SK, Thomason LC, Kuznetsov SG, Court DL. Recombineering: a homologous recombination-based method of genetic engineering. Nat Protoc. 2009;.

[2] Sawitzke JA, Costantino N, Li X, Thomason LC, Bubunenko M, Court C, et al. Probing cellular processes with oligo-mediated recombination and using the knowledge gained to optimize recombineering. J Molec Biol. 2011;407:45–59.

[3] Bos J, Zhang Q, Vyawahare S, Rogers E, Rosenberg SM, Austin RH. Emergence of antibiotic resistance from multinucleated bacterial filaments. Proc Natl Acad Sci. 2015;112(1):178–83.

[4] Zheng Q. Progress of a half century in the study of the Luria-Delbrück distribution. Math Biosci. 1999;162:1–32.

[5] Foster P. Methods for determining spontaneous mutation rates. Methods Enzymol. 2006;409:195–213.

[6] Zheng Q. Update on estimation of mutation rates using data from fluctuation experiments. Genetics. 2005;171:861–864.

[7] Zheng Q. Statistical and algorithmic methods for fluctuation analysis with SALVADOR as an implementation. Math Biosci. 2002;176:237–252.

[8] Hamon A, Ycart B. Statistics for the Luria-Delbrück distribution. Elec J Stat. 2012;6:1251–1272.

[9] Ycart B. Fluctation analysis: can estimates be trusted? PLoS One. 2013;8:e80958.

[10] Mazoyer A, Drouilhet R, Despréaux S, Ycart B. flan: An R package for inference on mutation models. The R Journal. 2017;9:334–351.

[11] Sarkar S, Ma WT, Sandri GvH. On fluctuation analysis: a new, simple and efficient method for computing the expected number of mutants. Genetica. 1992;85:173–179.

[12] Ryan FJ, Wainwright LK. Nuclear segregation and the growth of clones of spontaneous mutants of bacteria. J Gen Microbiol. 1954;11:364–379.

[13] Kondo S. A theoretical study on spontaneous mutation rate. Mutat Res. 1972;14:365–374.

[14] Koch AL. Mutation and growth rates from Luria-Delbrück fluctuation tests. Mutat Res. 1982;95:129–143.

[15] Kissling GE, Grogan DW, Drake JW. Confounders of mutation-rate estimators: selection and phenotypic lag in *Thermus thermophilus*. Mutat Res. 2013;749:16–20.

[16] Painter PR, Marr AG. Mathematics of microbial populations. Annu Rev Microbiol. 1968;22:519–548.

[17] Morreall J, Kim A, Liu Y, Degtyareva N, Weiss B, Doetsch PW. Evidence for retromutagenesis as a mechanism for adaptive mutation in *Escherichia coli*. PLoS Genet. 2015;11:e1005477.

[18] Wielgoss S, Barrick JE, Tenaillon O, Cruveiller S, Chane-Woon-Ming B, Médigue C, et al. Mutation rate inferred from synonymous substitutions in a long-term evolution experiment with *Escherichia coli*. G3. 2011;1:183–186.

[19] Jee J, Rasouly A, Shamovsky I, Akivis Y, Steinman SR, Mishra B, et al. Rates and mechanisms of bacterial mutagenesis from maximum-depth sequencing. Nature. 2016;534:693–696.

[20] Lee H, Popodi E, Tang H, Foster PL. Rate and molecular spectrum of spontaneous mutations in the bacterium *Escherichia coli* as determined by whole-genome sequencing. Proc Natl Acad Sci. 2012;109:E2774–E2783.

[21] Lind PA, Andersson DI. Whole-genome mutational biases in bacteria. PNAS. 2008;105:17878–17883.

[22] Bosshard L, Dupanloup I, Tenaillon O, Bruggmann R, Ackermann M, Peischl S, et al. Accumulation of deleterious mutations during bacterial range expansions. Genetics. 2017;207:669–684.

[23] Thomas P. Single-cell histories in growing populations: relating physiological variability to population growth; 2017.

[24] Otto SP, Whitton J. Polyploid incidence and evolution. Annu Rev Genet. 2000;34:401–437.

[25] Mode CJ. Multitype Branching Processes: Theory and Applications. New York: American Elsevier Publishing Company, Inc.; 1971.

[26] Uecker H, Hermisson J. The role of recombination in evolutionary rescue. Genetics. 2016;202:721–732.

[27] Martin G, Aguilée R, Ramsayer J, Kaltz O, Ronce O. The probability of evolutionary rescue: towards a quantitative comparison between theory and evolution experiments. Phil Trans Roy Soc B. 2013;368:20120088.

[28] Gerstein AC, Kuzmin A, Otto SP. Loss-of-heterozygosity facilitates passage through Haldane's sieve for *Saccharomyces cerevisiae* undergoing adaptation. Nat Commun. 2014;5:3819.

[29] Haldane JBS. A mathematical theory of natural and artificial selection, part V: selection and mutation. Proc Camb Philos Soc. 1927;28:838–844.

[30] Orr HA, Betancourt AJ. Haldane's Sieve and adaptation from the standing genetic variation. Genetics. 2001;157:875–884.
