# Supplementary material for: Effective polyploidy causes phenotypic delay and influences bacterial evolvability
Source: PLoS Biol. 2018 Feb 22;16(2):e2004644. doi: 10.1371/journal.pbio.2004644 (PMC5839593; doi:10.1371/journal.pbio.2004644)
Supplement: S1 Table — (DOCX) [file pbio.2004644.s002.docx]

| Target gene | Mutation | Phenotype | Expression profile | Fitness cost |
| --- | --- | --- | --- | --- |
| *rpoB* | H526L | RifR | Recessive[1] | 0.4±1.5%[2] |
| *gyrA* | D87G | NalR | Recessive[3] | 3.7±1.5%[2] |
| *rpsL* | K43R | StrepR | Recessive [3] | 0.5±1.4%[2] |
| *lacZ*(E461X) | X461E | lac+ | Dominant[4] | N/A |
| *YFP-*reporter | Correction of multiple nonsense mutations | | | N/A |

**S1 Table. List of mutations in this study.**

The point mutations in *rpoB*, *gyrA* and *rpsL* confer resistance to rifampicin (RifR), nalidixic acid (NalR) and streptomycin (StrepR), respectively[2]. The *lacZ*-system is based on repairing the nonsense mutation in a disrupted gene *lacZ*(E461X), restoring its ability to metabolize lactose[4]. Expression profiles were previously determined by co-expressing mutant and wild-type gene with one copy on the chromosome and another on a plasmid[1,3,4]. The *YFP*-reporter contains three stop codons (Y93X, Q95X, E96X, also see S2 Table) that were removed by recombineering in our single-cell microscopy experiment (main text, Fig 3).

**References**

1. Hayward RS. DNA Blockade by Rifampicin-Inactivated Escherichia coli RNA Polymerase, and Its Amelioration by a Specific Mutation. Eur J Biochem. 1976;71: 19–24.

2. Trindade S, Sousa A, Xavier KB, Dionisio F, Ferreira MG, Gordo I. Positive epistasis drives the acquisition of multidrug resistance. PLoS Genet. 2009;5: e1000578.

3. Edgar R, Friedman N, Molshanski-Mor S, Qimron U. Reversing bacterial resistance to antibiotics by phage-mediated delivery of dominant sensitive genes. Appl Environ Microbiol. 2012;78: 744–51.

4. Cupples CG, Miller JH. A set of lacZ mutations in Escherichia coli that allow rapid detection of each of the six base substitutions. Proc Natl Acad Sci. 1989;86: 5345–5349.
