# Supplementary figures and images for: Effective polyploidy causes phenotypic delay and influences bacterial evolvability
Source: PLoS Biol. 2018 Feb 22;16(2):e2004644. doi: 10.1371/journal.pbio.2004644 (PMC5839593; doi:10.1371/journal.pbio.2004644)

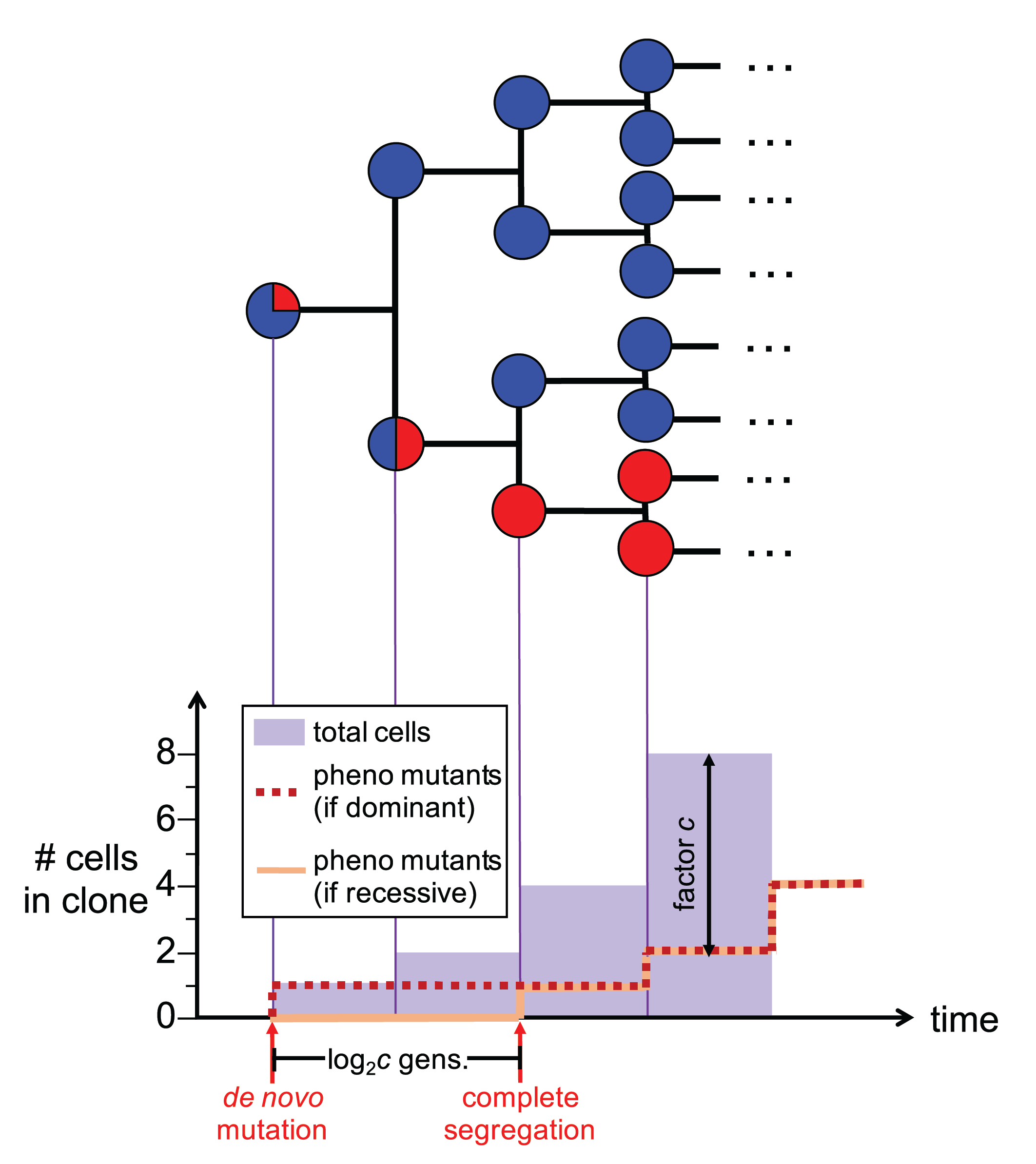

Supplement: S1 Fig — In this illustration, generation times are constant (synchronous division) for simplicity; ploidy c = 4 and a mutation arose in one copy. The existence of polyploidy implies that phenotypic mutants initially appear as singletons. For recessive traits, there is a delay of log2c generations after the de novo mutation until a single phenotypic mutant appears, then the number of phenotypic mutants doubles in each subsequent generation. For dominant traits, a single phenotypic mutant appears in the generation that the de novo mutation occurs but remains a singleton for log2c additional generations before the number of phenotypic mutants begins to double. In both cases, in the long term, a fraction 1/c of descendants are expected to be homozygous mutants and the rest wild-type. (TIF) [file pbio.2004644.s006.tif]

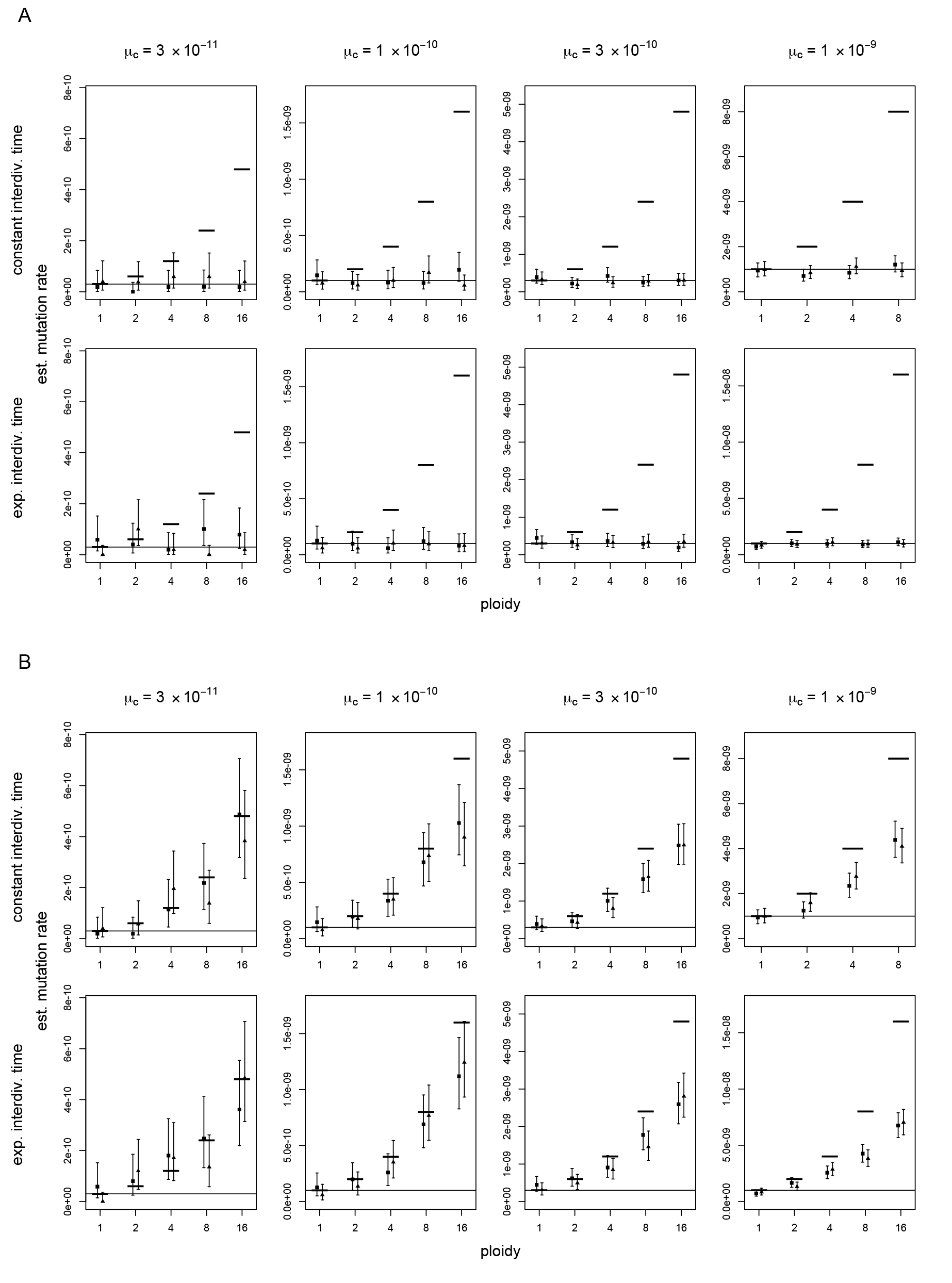

Supplement: S2 Fig — For various per-copy mutation rates μc (columns), for either constant (top row of each panel) or exponentially distributed (bottom row) interdivision times, and for each ploidy level c, 50 parallel cultures were simulated to make up one experiment. From each simulated culture, phenotypic mutants were counted assuming either (A) a recessive or (B) a dominant trait. This was repeated for two independent experiments (square and triangle symbols) for each parameter set. The MLE of mutation rate and 95% profile likelihood confidence intervals are plotted as a function of ploidy. In each panel, the lower solid black line indicates the actual per-copy mutation rate (μc) and the upper black dashes indicate the per-cell mutation rate (cμc). The results from the first simulated experiment with μc = 3 × 10^−10^ and constant interdivision times correspond to the main text Fig 6A and 6B. This figure can be reproduced using code and simulated data deposited on Dryad (http://dx.doi.org/10.5061/dryad.8723t). MLE, maximum likelihood estimate. (TIF) [file pbio.2004644.s007.tif]

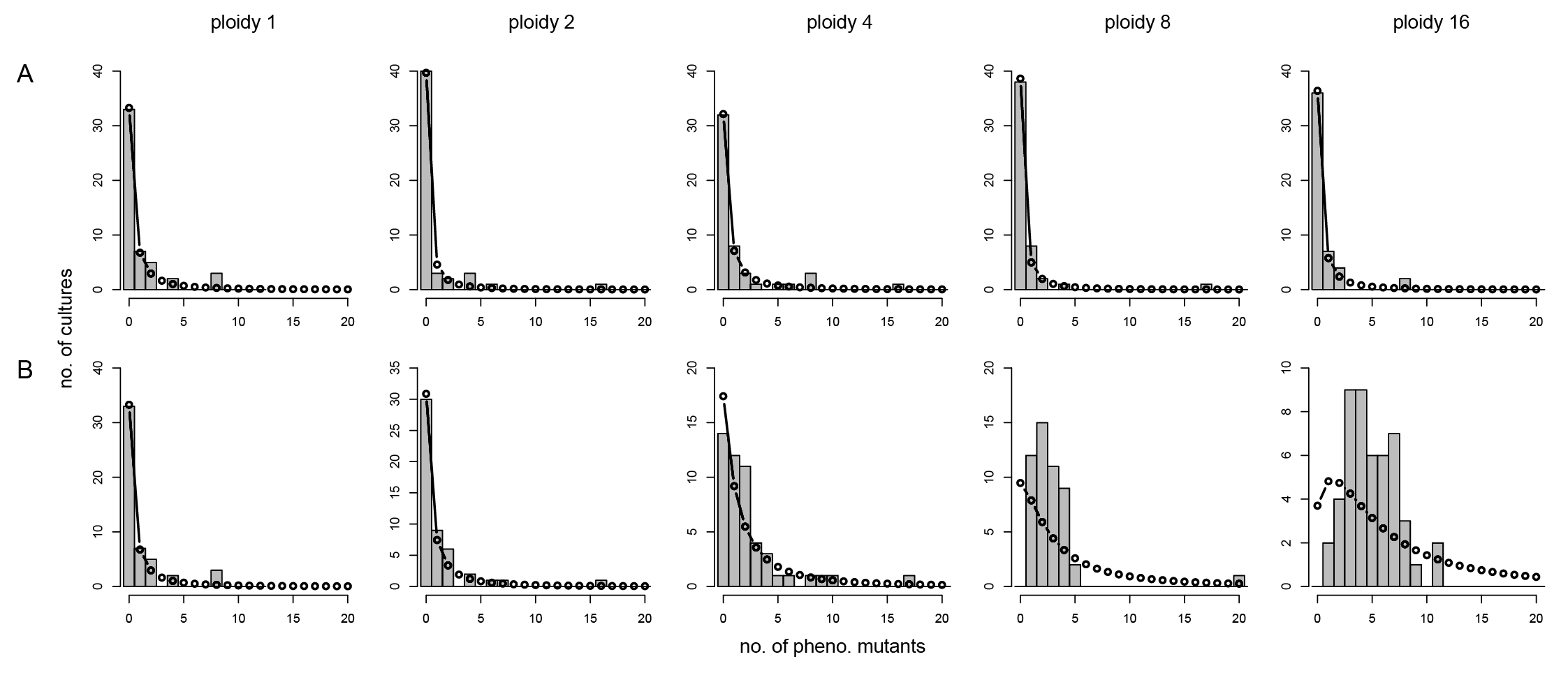

Supplement: S3 Fig — At each ploidy level, assuming the trait is either recessive (A) or dominant (B), the observed mutant count across 50 simulated parallel cultures is plotted as a histogram. The simulated data are the same as that used in S2 Fig for the first experiment with per-copy mutation rate μc = 3 × 10^−10^ and constant interdivision times. The distribution predicted by the standard model, parameterized by the maximum likelihood estimated mutation rate, is also plotted for comparison (connected points). The plots for ploidy c = 4 correspond to the main text Fig 6C and 6D. This figure can be reproduced using code and simulated data deposited on Dryad (http://dx.doi.org/10.5061/dryad.8723t). (TIF) [file pbio.2004644.s008.tif]

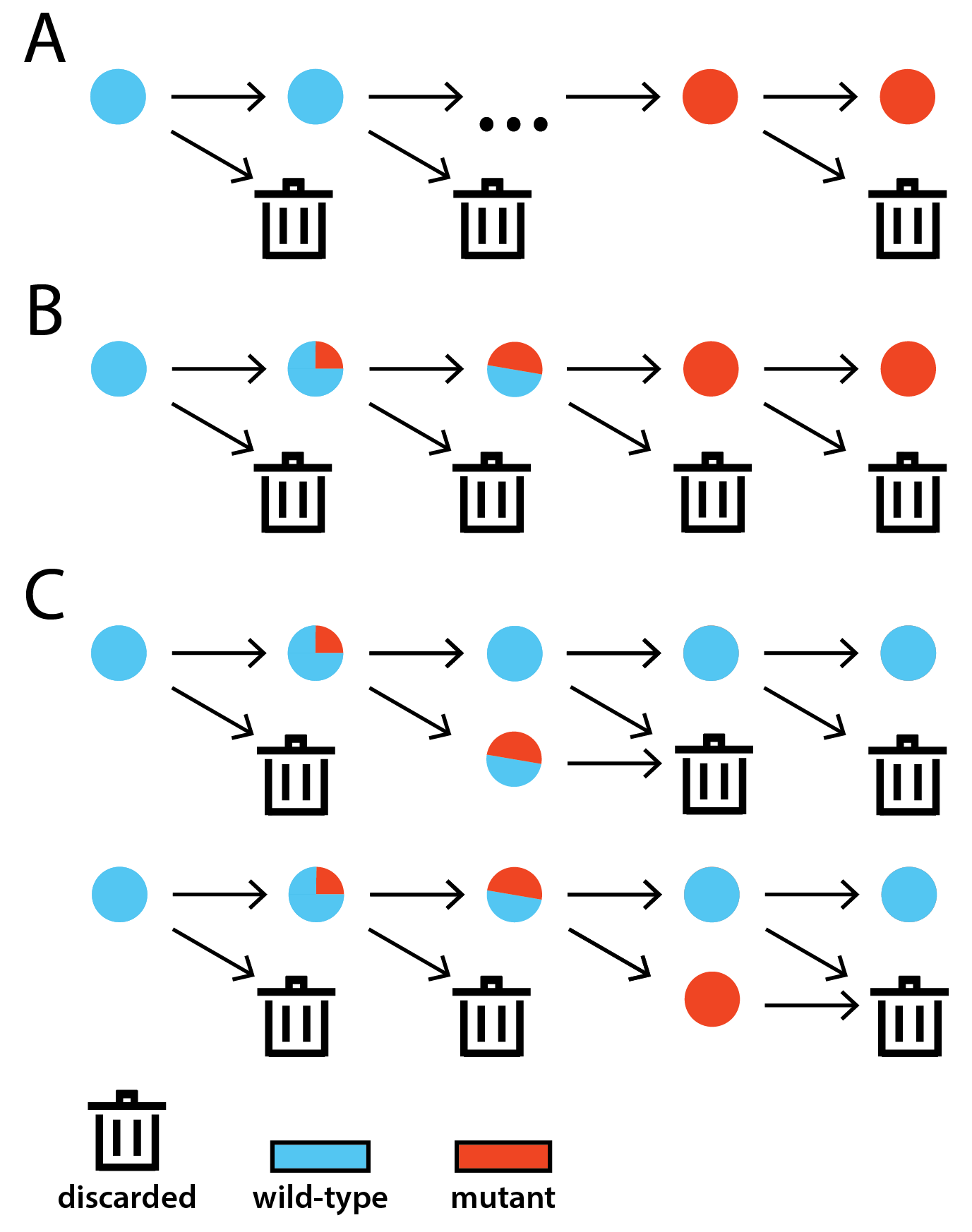

Supplement: S4 Fig — (A) In an MA experiment, a bacterial population evolves for thousands of generations with single-colony bottlenecking every 25 to 30 generations. However, we track only the lineage of direct ancestors leading to the ultimately sampled single cell. (B) In a polyploid cell, acquiring a mutation yields a heterozygous mutant cell. For this mutation to fix in the sampled lineage and be detected by WGS, the daughter cell inheriting the mutant copies must be chosen at each cell division for further propagation in the sampled lineage. (C) Asymmetric inheritance caused by polyploidy reduces the fixation probability of mutations because the daughter cell inheriting the mutation may not be sampled. MA, mutation accumulation; WGS, whole-genome sequencing. (TIF) [file pbio.2004644.s009.tif]

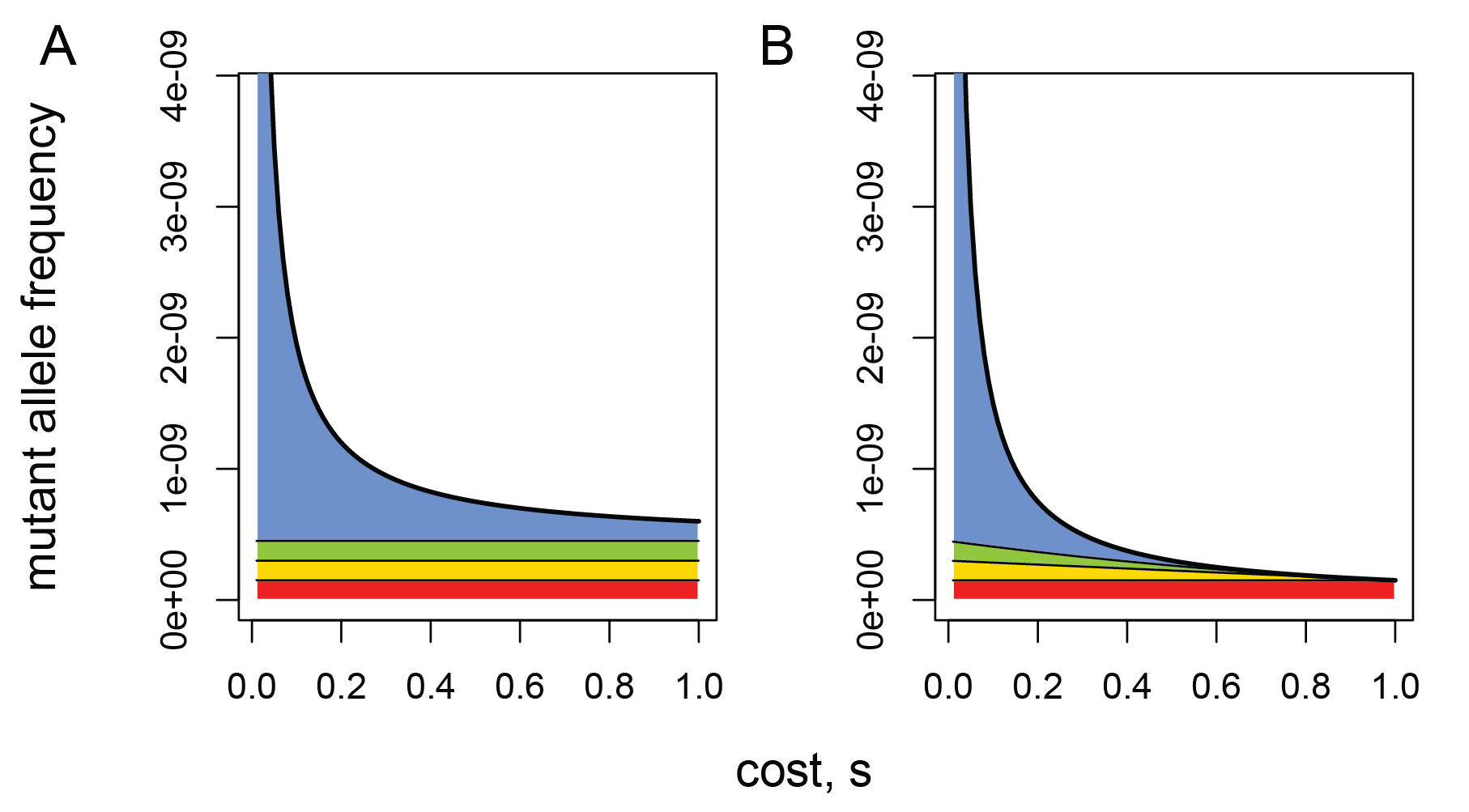

Supplement: S5 Fig — The frequency of the mutant allele is plotted as a function of its cost s, in the case of a completely recessive (A) or completely dominant (B) allele. In this example, ploidy c = 2n = 8 and per-copy mutation rate μ˜c=3×10^−10^. The contribution to this frequency made by each cell type (given by (2i/2n)x2i* in cells containing 2i mutant chromosomes) is represented by the shaded area between two curves, working up from the bottom through heterozygotes (0 ≤ i ≤ n-1) and then homozygotes (i = n). This figure can be reproduced using code deposited on Dryad (http://dx.doi.org/10.5061/dryad.8723t). (TIF) [file pbio.2004644.s010.tif]

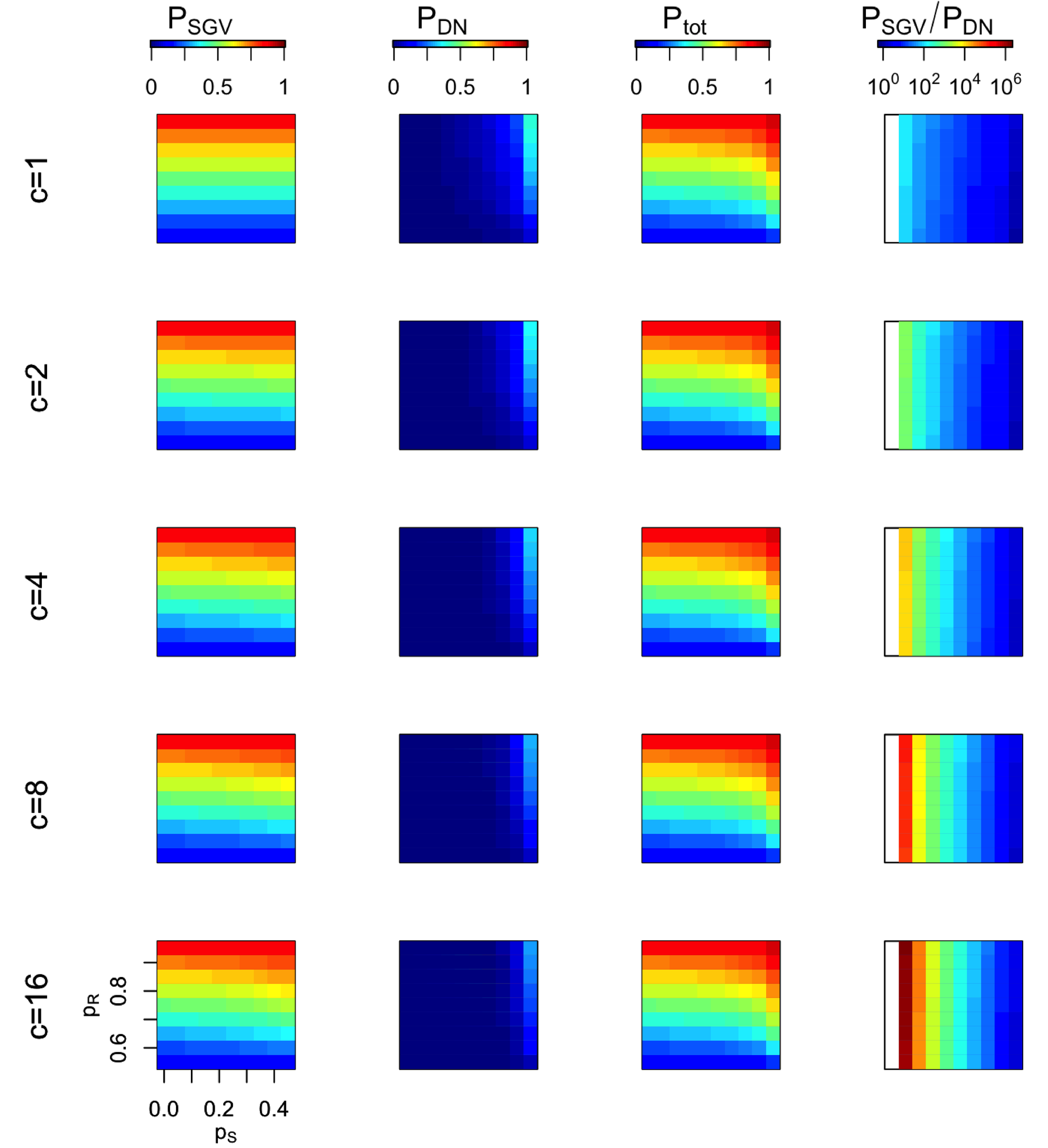

Supplement: S6 Fig — From left to right: probability of rescue from SGV, PSGV; from de novo mutations, PDN; from either or both, Ptot; and the ratio of probabilities, PSGV/PDN (on log scale). The missing values (white) occur where PDN = 0 and the ratio is undefined. All quantities are plotted as functions of the probabilities of division before death of sensitive cells (pS; horizontal axis) and resistant cells (pR; vertical axis). Ploidy (c) varies by row as indicated. Additional model parameters s = 0.1 and m = 0.06 are fixed. This figure can be reproduced using code deposited on Dryad (http://dx.doi.org/10.5061/dryad.8723t). SGV, standing genetic variation. (TIF) [file pbio.2004644.s011.tif]

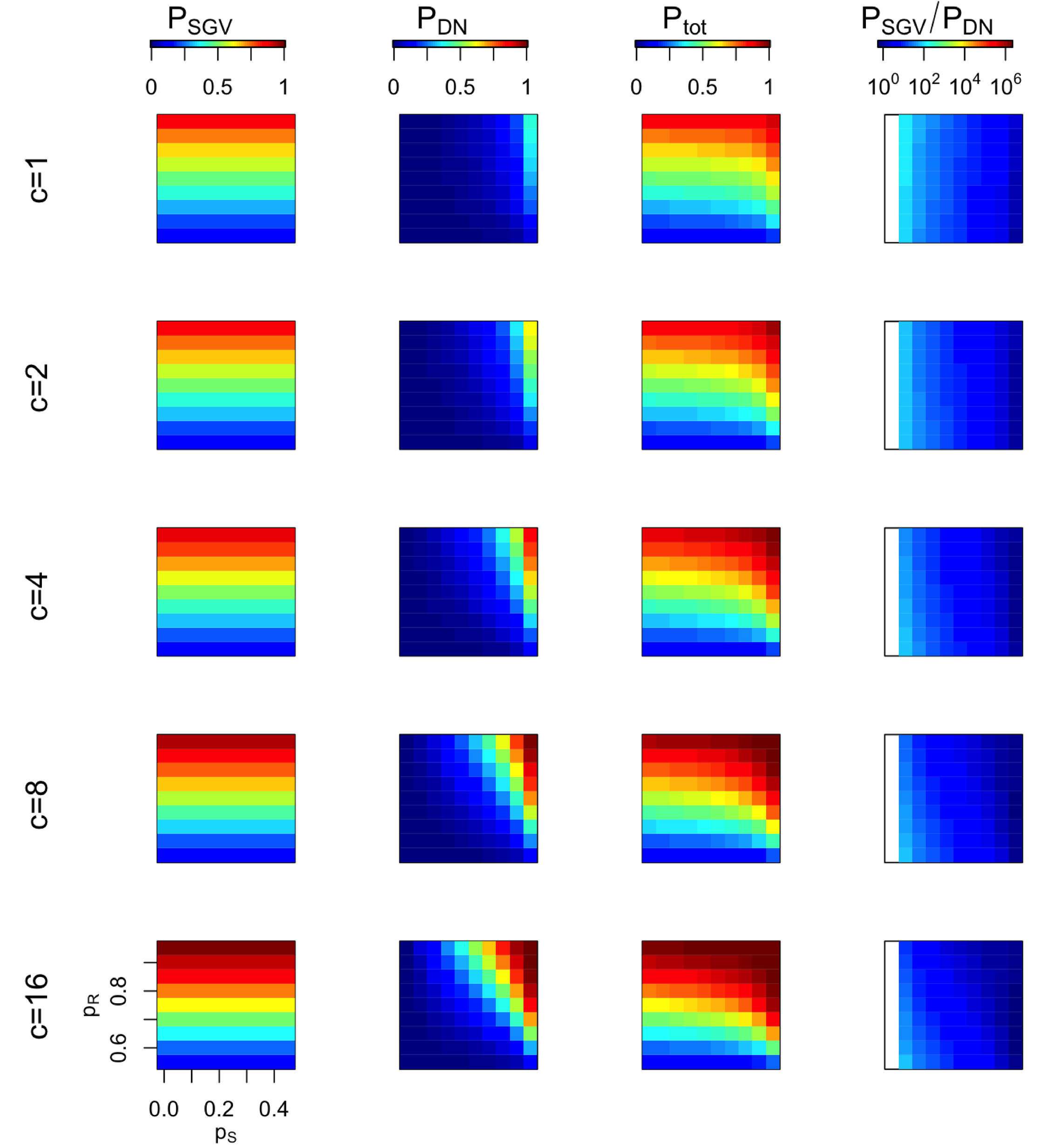

Supplement: S7 Fig — All plotting parameters are identical to S6 Fig. This figure can be reproduced using code deposited on Dryad (http://dx.doi.org/10.5061/dryad.8723t). (TIF) [file pbio.2004644.s012.tif]

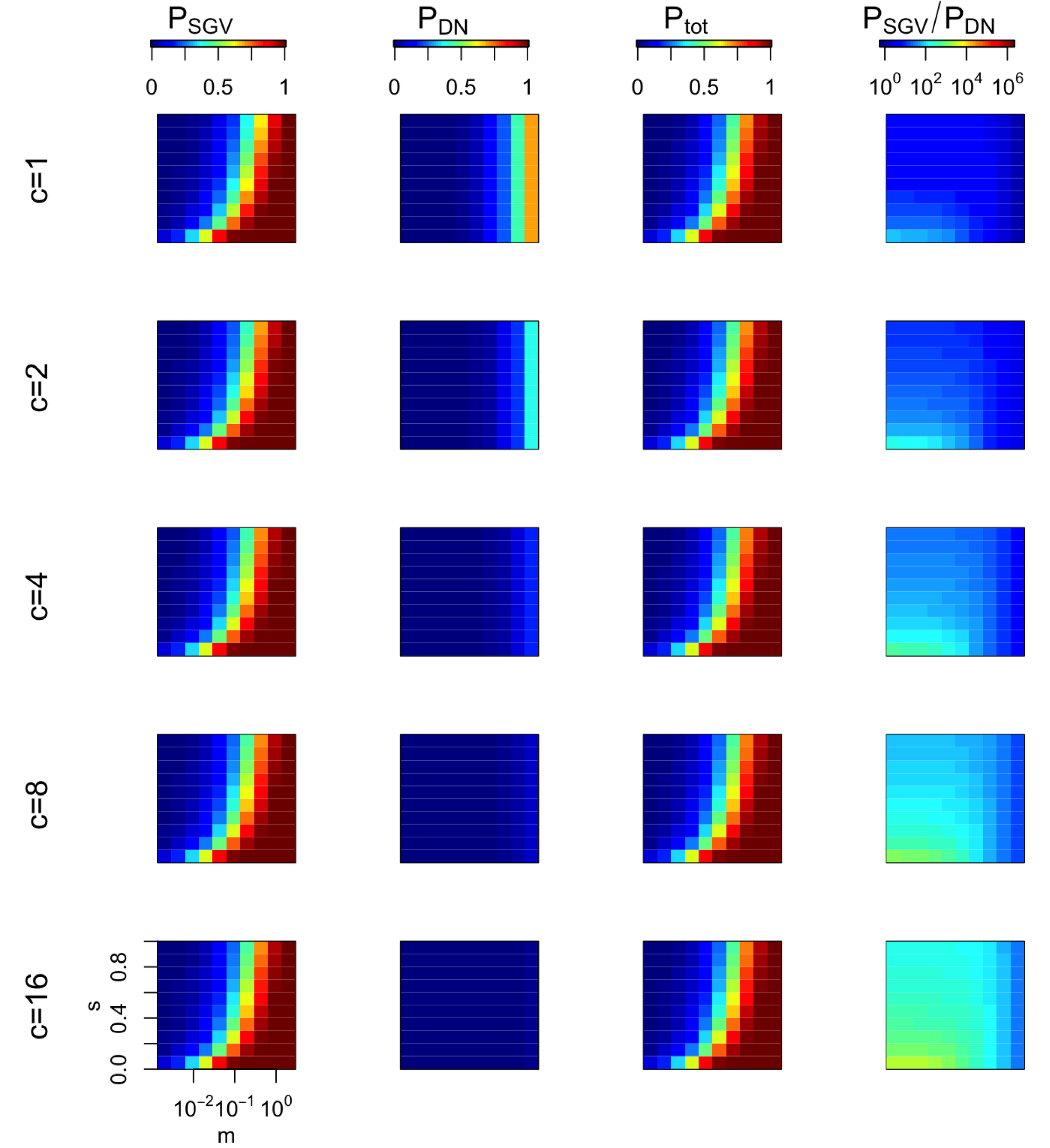

Supplement: S8 Fig — From left to right: probability of rescue from SGV, PSGV; from de novo mutations, PDN; from either or both, Ptot; and the ratio of probabilities, PSGV/PDN (on log scale). All quantities are plotted as functions of the base-10 log of mutational influx (log10(m) = log10(Nμ∼c); horizontal axis in each plot) and the cost of the mutation in the old environment (s; vertical axis in each plot). Ploidy (c) varies by row as indicated. Additional model parameters pS = 0.2 and pR = 0.9 are fixed. This figure can be reproduced using code deposited on Dryad (http://dx.doi.org/10.5061/dryad.8723t). SGV, standing genetic variation. (TIF) [file pbio.2004644.s013.tif]

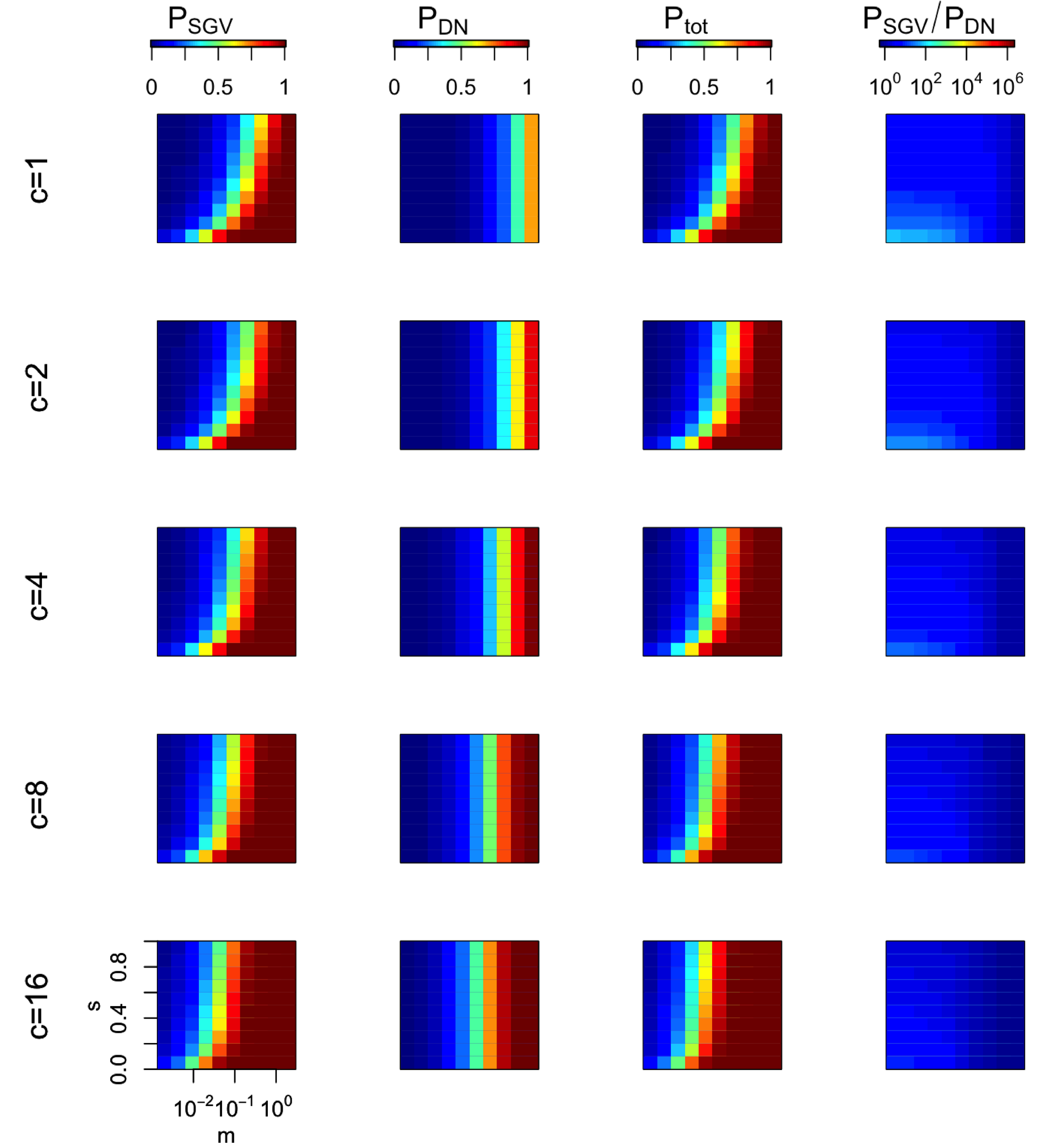

Supplement: S9 Fig — All plotting parameters are identical to S8 Fig. This figure can be reproduced using code deposited on Dryad (http://dx.doi.org/10.5061/dryad.8723t). (TIF) [file pbio.2004644.s014.tif]

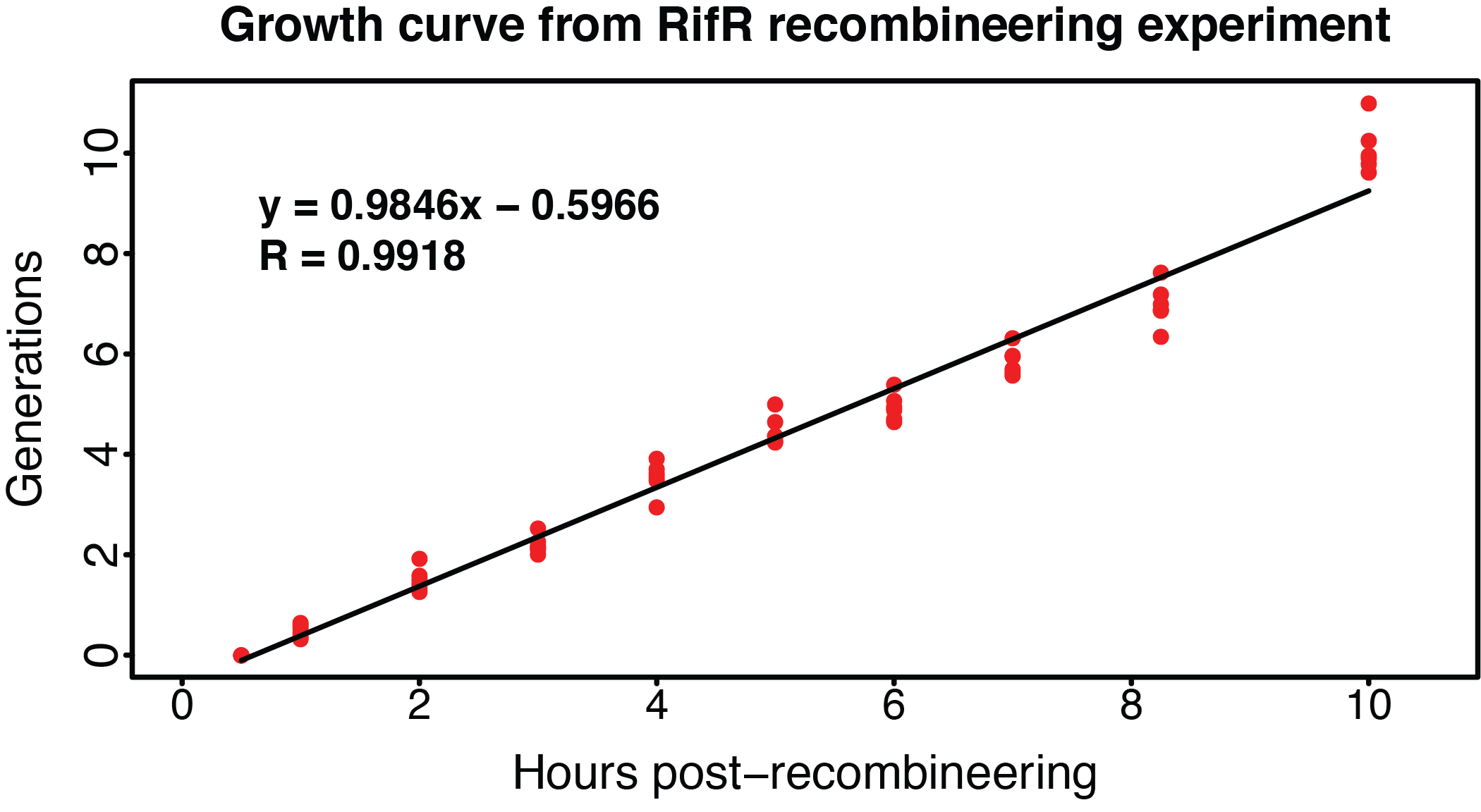

Supplement: S10 Fig — The illustrated growth curve is from the RifR recombineering experiment. Sampling occurred during the first 10 hours after recombineering, with the first sample taken at 0.5 h immediately after recovery from electroporation. Population doublings, expressed in number of generations after the first sampling time point, is calculated based on CFU counts at each sampling time. Red dots indicate individual data points from each of six replicates sampled at each time. The black line shows a linear regression of how generation time depends on real time expressed in hours, fitted through the mean of the replicates at each time point. The numerical values can be found in S1 Data. CFU, colony-forming unit. (TIF) [file pbio.2004644.s015.tif]
